# Analysis of agreement between measures of subjective cognitive impairment and probable dementia in the National Health and Aging Trends Study

**DOI:** 10.1002/alz.13758

**Published:** 2024-03-01

**Authors:** Linda C. Chyr, Jennifer L. Wolff, Julie M. Zissimopoulos, Emmanuel F. Drabo

**Affiliations:** ^1^ Enterprise Analytics Core, Elevance Health, Inc. Wilmington Delaware USA; ^2^ Department of Health Policy and Management John Hopkins Bloomberg School of Public Health Baltimore Maryland USA; ^3^ Sol Price School of Public Policy University of Southern California Los Angeles California USA; ^4^ Leonard D. Schaeffer Center for Health Policy University of Southern California Los Angeles California USA

**Keywords:** ADRD, Alzheimer's disease, dementia, disability questionnaire, NHATS, subjective cognitive impairment

## Abstract

**BACKGROUND:**

Subjective cognitive impairment (SCI) measures in population‐based surveys offer potential for dementia surveillance, yet their validation against established dementia measures is lacking.

**METHODS:**

We assessed agreement between SCI and a validated probable dementia algorithm in a random one‐third sample (*n* = 1936) of participants in the 2012 National Health and Aging Trends Study (NHATS).

**RESULTS:**

SCI was more prevalent than probable dementia (12.2% vs 8.4%). Agreement between measures was 90.0% and of substantial strength. Misclassification rates were higher among older and less‐educated subgroups due to higher prevalence of false‐positive misclassification but did not vary by sex or race and ethnicity.

**DISCUSSION:**

SCI sensitivity (63.4%) and specificity (92.5%) against dementia were comparable with similar metrics for the NHATS probable dementia measure against the “gold‐standard” Aging, Demographics, and Memory Study–based dementia criteria, implying that population‐based surveys may afford cost‐effective opportunities for dementia surveillance to assess risk and inform policy.

**Highlights:**

- The prevalence of subjective cognitive impairment (SCI) is generally higher than that of a validated measure of probable dementia, particularly within the youngest age group, females, Whites, and persons with a college or higher degree.
- Percent agreement between SCI and a validated measure of probable dementia was 90.0% and of substantial strength (prevalence‐ and bias‐adjusted kappa, 0.80). Agreement rates were higher in older and less‐educated subgroups, driven by the higher prevalence of false‐positive disagreement, but did not vary significantly by sex or race and ethnicity.
- SCI's overall sensitivity and specificity were 63.4% and 92.5%, respectively, against a validated measure of probable dementia, suggesting utility as a low‐cost option for dementia surveillance. Heterogeneity in agreement quality across subpopulations warrants caution in its use for subgroup analyses.

## 1 BACKGROUND

Surveillance is crucial for detecting patterns, understanding risk factors and health conditions, and identifying intervention opportunities in public health. However, a robust dementia surveillance system, like the national registry for cancer surveillance, is lacking in the United States for various reasons, including the lack of cost‐effective biomarkers for dementia identification, failure to implement routine dementia assessment,^1^, ^2^ and methodological challenges and biases in widely used data.^3^ As the number of older adults (≥65 years) living with Alzheimer's disease and related dementias (ADRD; dementia henceforth) is projected to triple by 2050 from its current level of 6.2 million,^4^ establishing surveillance systems to monitor the health and care needs of this growing population will be critical to designing effective local, state and national policy, and informing health care system organization and clinical practice. By one estimate, AD diagnosis in its early stages could save over $7 trillion in health and long‐term care costs.^4^ Ongoing, timely, and systematic collection and analysis of information is foundational to effective surveillance, and several data sources now available could be used in dementia surveillance.

Subjective cognitive impairment (SCI) is a core component of the diagnostic criteria for mild cognitive impairment (MCI),^5^ one of the early manifestations of dementia.^4^, ^6^, ^7^, ^8^, ^9^, ^10^, ^11^, ^12^ SCI describes the occurrence of a person reporting or admitting to impaired cognitive function.^12^, ^13^ SCI measures self‐perception of cognitive performance, and is conceptually independent of normal aging, performance on a cognitive test, or clinical diagnosis.^12^, ^14^ The Medicare Annual Wellness Visit (AWV), introduced in 2011, relies on self‐reported concerns analogous to SCI, followed by direct assessment of cognitive function, as a condition of reimbursement, thus potentially incorporating SCI as a part of routine screening.^15^, ^16^, ^17^ SCI is also fielded in several major national population‐based surveys, including the American Community Survey (ACS), the Behavioral Risk Factor Surveillance System (BRFSS), and the National Health Interview Survey (NHIS) (see Supplementary Materials).^18^, ^19^, ^20^, ^21^ Since 2010, these surveys have incorporated the U.S. Census Bureau's standardized Disability Questionnaire, consisting of six items assessing disability status along the domains of hearing, vision, cognition, ambulation, self‐care, and independent living. These items represent a minimum standard for inclusion in population surveys on disability^22^, ^23^ and afford opportunities to monitor, study, and inform policies about disabilities.^24^

The cognitive disability item of the Disability Questionnaire asks respondents whether they have difficulty remembering, concentrating, or making decisions because of a physical, mental, or emotional problem. Although surveys differ in design, sample size, and data collection methods and periods, question wording is nearly identical (eTable S1). This item, when combined with available local‐ and individual‐level data, could facilitate monitoring trends in cognitive impairment across subpopulations and communities, the detection of potential “hot spots” and disparities, and understanding of local needs and resources that could be rationally and equitably leveraged or redirected to maximize impact.

Although SCI is now used in local dementia care—Washington State already uses the American Community Survey's (ACS's) SCI measure for dementia monitoring and resource needs planning^25^—its validity as a dementia surveillance tool and how it applies to different subpopulations remains unknown. This study fills this critical gap in knowledge by assessing and interpreting the performance of SCI against a validated measure of probable dementia, both fielded in a special module one‐third random sample of participants—of round 2 of the National Health and Aging Trends Study (NHATS) and that serves as a reference standard to assess the validity of SCI in detecting dementia risk in the population.

Given that not all SCI is indicative of dementia, we first hypothesize that the prevalence of SCI will be higher than that of dementia. Our second hypothesis is that SCI will have agreement with dementia that is at least moderate in strength. Thus, we anticipate that the sensitivity and specificity of SCI against dementia will align closely with those of the NHATS probable dementia definition against the “gold‐standard” Aging, Demographics, and Memory Study (ADAMS)–based criteria for dementia and normal plus cognitive impairment with no dementia.

## 2 METHODS

### 2.1 Study design, population, and data source

NHATS is a nationally representative longitudinal survey of Medicare beneficiaries, conducted annually since 2011, that is designed to study trends and dynamics in late‐life functioning.^26^ NHATS participants undergo annual assessments of their sociodemographic, socioeconomic, functional, and health status, and receipt of medical and non‐medical care.^27^ We draw on a special module of the 2012 NHATS in which a random one‐third sample of NHATS participants (*n* = 1936) were administered the U.S. Census Bureau's Disability Questionnaire.^28^

### 2.2 Measures

#### 2.2.1 Probable dementia

Probable dementia status was operationalized using a validated composite measure developed from information collected in NHATS, including reported by self‐ and proxy‐respondents as well as scores on the Eight‐item Interview to Differentiate Aging and Dementia (AD8) or cognitive tests of memory, orientation (modified telephone interview for cognitive status, TICSm),^29^ and executive function (Clock‐Drawing Test, CDT).^30^ Individuals were classified as having probable dementia on the basis of meeting one of the following three criteria: (1) a self‐ or proxy‐respondent's report of a doctor's diagnosis of dementia or Alzheimer's disease; (2) a score indicating likely dementia (i.e., a score of 2 or higher) on the AD8, a validated proxy‐report assessment of dementia^31^, ^32^, ^33^; or (3) impairment based on scoring at or below 1.5 standard deviations (SDs) from the sample mean score in at least two of three cognitive domains from test items that evaluated the person's memory (immediate and delayed 10‐word recall), orientation (date, month, year, and day of the week; naming the President and Vice President), and executive functioning.^30^, ^34^, ^35^, ^36^ The following uniform cutoffs were used for each cognitive domain: ≤3 for memory (scale, 0–20), ≤3 for orientation (scale, 0–8), and ≤1 for executive functioning (scale, 0–5). These tests were administered to all self‐respondents and approximately half of proxy‐respondents. Test scores were also used for self‐respondents who did not report having a prior diagnosis of dementia, as well as for consenting sample participants with proxy‐respondents reporting no doctor's diagnosis of ADRD, and no behavior change consistent with dementia on the AD8 in their sample participant (Supplementary Materials, Section 2).^30^ The NHATS probable dementia definition was validated against the ADAMS diagnostic classifications for dementia,^30^ and is considered the reference standard for dementia in this analysis.

#### 2.2.2 Subjective cognitive impairment

We defined SCI status from responses to the cognitive disability item of the Disability Questionnaire, which asks the following (ACS version): *“Because of a physical, mental, or emotional problem, does this person have difficulty remembering, concentrating, or making decisions?”* ^37^, ^38^ Respondents who affirmatively answered this question were categorized as having SCI; those who answered “No” were classified as not having SCI; all other respondents (e.g., those with missing answers, or who answered “Don't know/not sure”) were classified as missing responses (unweighted *n* = 18) and excluded from the analysis. The excluded respondents were significantly more likely to have been hospitalized in the past year, be underweight, and less likely to be obese, but were otherwise similar in all other observable characteristics to those with affirmative or negative responses (eTable S2).

**RESEARCH IN CONTEXT**

1. **Systematic review**: The cognitive disability item of the U.S. Census Bureau's standardized Disability Questionnaire, now routinely included in several large population‐based surveys, asks respondents to report experience of difficulty with memory, concentration, and decision‐making. Although subjectively reported cognitive impairments are a marker of dementia risk, the cognitive disability item of the Disability Questionnaire has not been evaluated previously against validated measures of dementia.
2. **Interpretation**: We evaluated the subjective cognitive impairment (SCI) measure against a validated algorithm of probable dementia status from the National Health and Aging Trends Study to assess SCI's utility for dementia surveillance in the United States.
3. **Future directions**: Routine monitoring of SCI and similar measures less reliant on multiple cognitive function questions in population‐based surveys may afford low‐cost opportunities for dementia risk surveillance to better understand patterns in dementia risk, impacts and burden across jurisdictions, communities, and diverse populations, and inform effective dementia policies.

#### 2.2.3 Agreement between measures

We constructed a dummy variable taking the value of 1 to indicate agreement between each respondent's SCI and probable dementia status, defined as situations where the respondent was classified as having both SCI and dementia or having neither (i.e., a+d; eTable S3); all other situations were defined as disagreement (i.e., b+c) and coded as zero.

#### 2.2.4 Other covariates

Other covariates included measures reflecting established risk factors for cognitive impairment and dementia, such as age, sex, race and ethnicity, and educational attainment. Details on the construction of the covariates are provided in Supplementary Materials, Section 4.

### 2.3 Statistical analyses

We compared the characteristics of NHATS participants assessed for SCI in 2012 by dementia status. Pearson's chi‐square tests were used to compare binary and categorical variables, and student's *t*‐tests were used to compare means for continuous variables. We calculated dementia prevalence by SCI status, as well as SCI prevalence by dementia status, overall, and by age groups, sex, race and ethnicity, and education.

#### 2.3.1 Agreement between measures

We calculated the Cohen and Conger's kappa statistic (κ),^39^, ^40^ a measure of the proportion of agreement greater than that expected by chance, used to assess the strength of agreement between the measures (Equation S1). The standard range of κ is 0 for no agreement and 1 for complete agreement, although values from −1 to 0 are possible and would indicate negative correlation.^39^, ^40^ A higher value of κ indicates, therefore, stronger agreement between measures.^39^, ^40^ To calculate kappa, we first defined and calculated agreement of SCI with probable dementia as the percentage of respondents with an observed agreement between the SCI and probable dementia status (Equation S2). Second, we calculated the expected agreement between SCI and probable dementia, a statistic that captures how much agreement would be expected to exist due to chance alone (Equation S3).

In a 2 by 2 table where the marginal totals are relatively balanced, the Cohen and Conger's kappa statistic alone is an appropriate measure of reliability. However, when there is imbalance in the marginal totals, the kappa statistic alone does not always describe sufficiently the level of reliability, as its magnitude is influenced by other factors such as disease prevalence, bias, and non‐independence of ratings, which may complicate its interpretation and result in two paradoxes: the prevalence and bias paradoxes (Supplementary Materials, Section 5.1.2).^41^, ^42^ The former paradox arises from the fact that when the prevalence of a given rating in the population is very high or low (that is, when the expected agreement between measures is high), even a relatively high value of the observed agreement may produce a low value of kappa. The latter paradox is a consequence of the fact that “unbalanced marginal totals produce higher values of kappa than more balanced totals.”^41^

To account for these effects, we, therefore, calculated the prevalence‐ and bias‐adjusted kappa (PABAK, $\kappa^{*}$), which adjusts the Cohen and Conger's kappa for imbalances caused by differences in the prevalence and bias (Supplementary Materials, Section 5.1.2).^42^ The PABAK's mathematical expression, is $\kappa^{*}=\;2p_{o}-1$. All results are interpreted using the values of the PABAK. We used the Landis and Koch benchmark scale to interpret these estimated measures of the strength of agreement between measures (eTable S4).^43^ For brevity and clarity of exposition, results are interpreted using the PABAK.

#### 2.3.2 Individual characteristics associated with disagreement between measures (misclassification)

We used logistic regression models to examine the associations between established dementia risk factors and binary indicators of misclassification overall as well as by false‐positive and false‐negative disagreement between measures. Because the odds ratios from the logistic regression model can dramatically overestimate the prevalence ratio when the outcome prevalence is high (≥ 10%),^44^, ^45^ we also estimated log‐binomial,^46^ Cox's proportional hazard,^47^, ^48^ and modified (robust) Poisson models.^49^ We first note that in addition to helping identify predictors of misclassification, these models help correct for the prevalence and bias paradoxes. The standard errors of the estimated parameters from the log‐binomial models will also be generally smaller than those from the standard logistic regression models. Finally, due to the cross‐sectional nature of the data, with measurements occurring within a single year, the estimated hazard ratios from the Cox's proportional hazard model will be identical to prevalence ratios from the modified Poisson models. For brevity and ease of exposition, our results section focuses on estimates from the log‐binomial models, and we briefly comment on similarities and differences between estimates from the log‐binomial and other specifications.

#### 2.3.3 SCI's accuracy in detecting probable dementia

To further evaluate SCI's accuracy in detecting probable dementia, we calculated two statistics: (1) sensitivity (i.e., true positive rate), which measures the ability of SCI to correctly identify individuals with the NHATS validated algorithm of probable dementia status; and (2) specificity (i.e., true negative rate), which assesses SCI's ability to correctly detect those without probable dementia. For excellent overviews of these metrics, see Trevethan ( 2017) and Grunau and Linn (2018).^50^, ^51^, ^52^

#### 2.3.4 Robustness analyses

Additional accuracy statistics that were calculated are presented in the Supplementary Materials, including (1) positive predictive value (PPV), which measures whether those who are assessed as having SCI also have probable dementia; and (2) negative predictive value (NPV), which measures whether those assessed as not having SCI also do not have probable dementia. We constructed these statistics overall and by age group, sex, race and ethnicity, and education.

As the accuracy statistics described above may vary with disease prevalence,^52^, ^53^ we also calculated other statistics that are invariant with prevalence, including: (1) likelihood ratios; (2) odds ratios (ORs); and (3) the area under the receiver‐operating characteristic (ROC) curve (AUC), which is interpreted using cutoffs such as those proposed by Hosme and Lemeshow (2000) ^54^, ^55^ (eTable S5). These statistics, collectively, help further characterize the accuracy of SCI against our validated measure of probable dementia. Details on the definitions, calculations, and interpretation of these metrics are summarized in the Supplementary Materials, Sections 6‐7.

All analyses were conducted in Stata 16.1,^56^ and used NHATS Round 2 analytic weights to produce a nationally representative sample and account for differential probabilities of selection and nonresponse,^27^, ^28^, ^57^ and were adjusted to reflect that the analytic sample is a random one‐third sample of NHATS participants in 2012.

## 3 RESULTS

The sample included *n* = 1936 respondents (weighted sample size, *n* = 35,489,497; Table 1). Respondents with probable dementia and possible dementia differed from those without dementia along all characteristics (Table 1).

**TABLE 1:** Characteristics of older adults with and without NHATS probable dementia in 2012, who participated in the disability questionnaire.

Characteristics	Total	No dementia^c^	Possible dementia	Probable dementia^c^	*p*‐value^d^
**Overall**					
Unweighted, *N*	1936	1322	397	217	<0.001
% of total	–	68.3	20.5	11.2	
Weighted^a^, *N*	35,489,497	26,580,233	5,910,211	2,999,053	<0.001
% of total	–	74.9	16.7	8.5	
**SCI, *n* (%)**	289 (12.2)	74 (5.1)	77 (18.2)	138 (63.5)	<0.001
**Age (y), mean (SD)**	76.2 (7.2)	74.8 (6.1)	79.4 (8.5)	81.6 (8.7)	<0.001
**Age groups (y), *n* (%)**					
65–74	696 (48.9)	582 (56.3)	89 (31.0)	25 (18.9)	<0.001
75–84	777 (35.7)	527 (33.6)	164 (40.2)	86 (44.5)	
≥85	463 (15.4)	213 (10.1)	144 (28.7)	106 (36.6)	
**Female, *n* (%)**	1132 (56.7)	777 (56.9)	213 (51.8)	142 (65.3)	0.041
**Race and ethnicity, *n* (%)**					<0.001
White, non‐Hispanic	1341 (81.4)	989 (85.7)	231 (69.4)	121 (67.5)	
Black, non‐Hispanic	408 (8.1)	237 (6.8)	109 (11.8)	62 (12.1)	
Hispanic	114 (6.5)	58 (4.6)	36 (12.3)	20 (12.2)	
Other, non‐Hispanic	73 (4.0)	38 (2.9)	21 (6.5)	14 (8.2)	
**Education, *n* (%)**					<0.001
Less than high school	496 (20.4)	227 (14.1)	166 (36.9)	103 (43.1)	
High school graduate	658 (35.4)	465 (35.8)	124 (34.1)	69 (34.6)	
Some college	336 (18.2)	261 (19.8)	53 (14.2)	22 (11.9)	
College graduate and higher	446 (26.0)	369 (30.2)	54 (14.8)	23 (10.4)	
**Residential care not nursing home** ^b^, ***n*(%)**	116 (5.6)	53 (3.7)	34 (9.5)	29 (14.8)	<0.001
**Proxy respondent, *n* (%)**	131 (5.0)	10 (0.7)	19 (3.7)	102 (45.6)	<0.001

^a^ Initial sample consists of a random one‐third sample of NHATS participants in 2012. Data are presented as unweighted frequencies (weighted % for categorical measures), and weighted mean (weighted SD) for continuous measures. Percentages are of column total and are survey‐weighted, except when noted otherwise. Weighting was done using round 2 analytic weights to produce a nationally representative sample and account for differential probabilities of selection and nonresponse in NHATS, and were further adjusted to reflect that the analytic sample is a random one‐third sample of NHATS participants in 2012.

Abbreviations: NHATS, National Health and Aging Trends Study; SCI, subjective cognitive impairment; SD, standard deviation; y, years.

^b^ Respondents residing in a nursing home residential care setting in round 1 or round 2 were excluded from this analysis. Hence, the sample includes only older adults residing in the community or in a residential care setting other than a nursing home.

^c^ No dementia includes NHATS categorization of possible dementia.

^d^ Determined by Pearson's chi‐square test for binary and categorical variables and Student's *t*‐test for continuous variables.

### 3.1 SCI and probable dementia prevalence

**FIGURE 1:**
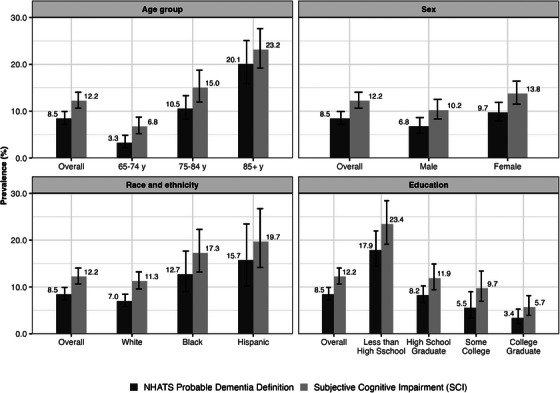
Prevalence of NHATS probable dementia and SCI among U.S. older adults in 2012, by age group, sex, race and ethnicity, and education. NHATS, National Health and Aging Trends Study; SCI, subjective cognitive impairment; y, years. Notes: Dementia denotes NHATS probable dementia. All estimates are survey‐weighted; the bars represent the 95% confidence interval around the point estimates, denoted by the numbers next to each bar. All estimates are unadjusted for individual characteristics. Other race and ethnicity category is not reported due to the small sample size (unweighted *n* = 73). Numerical estimates are provided in eTable S6. Sample included *n* = 1863 observations (weighted *n* = 34,076,757). All estimates are adjusted using round 2 analytic weights, to produce a nationally representative sample and account for differential probabilities of selection and nonresponse in NHATS and were further adjusted to reflect that the analytic sample is a random one‐third sample of NHATS participants in 2012.

Overall, SCI prevalence was 12.2% (95% CI 10.6–14.0) and the prevalence of probable dementia was 8.5% (95% CI 7.2–9.9; Figure 1 and eTable S6). Prevalence for possible or probable dementia (combined) was 25.1% (95% CI 22.2–28.2; Table 1 and eTable S6).

Within subgroups, and across measures, the prevalence of SCI was statistically significantly higher than that of probable dementia in the youngest age group (SCI: 6.8%, 95% CI 5.2–8.7 vs dementia: 3.3%, 95% CI 2.2–4.9), among females (SCI: 13.8%, 95% CI 11.5–16.4 vs dementia: 9.7%, 95% CI 7.9–11.9), Whites (SCI: 11.3%, 95% CI 9.6–13.2 vs dementia: 7.0%, 95% CI 5.8–8.4), and those with some college degree (SCI: 9.7%, 95% CI 7.0–13.4 vs dementia: 5.5%, 95% CI 3.4–9.0). The prevalence of SCI and probable dementia did not differ significantly in older age groups (75–84 years and ≥85 years) or among males, Blacks, Hispanics, and individuals with educational attainment other than some college degree.

Across subgroups, patterns of SCI and probable dementia prevalence were, for the most part, consistent with findings in prior studies. The prevalence of SCI and probable dementia were higher in older than younger age groups. Prevalence rates of both SCI and probable dementia did not differ between men and women. SCI prevalence rates were similar for Blacks and Hispanics, and higher than rates for Whites (Blacks: 17.3%, 95% CI 13.2–22.3 vs Hispanics: 19.7%, 95% CI 14.2–26.6 vs Whites: 11.3%, 95% CI 9.6–13.2). Likewise, probable dementia prevalence rates were similar for Blacks and Hispanics (Blacks: 12.7%, 95% CI 9.0–17.6 vs Hispanics: 15.7%, 95% CI 10.3–23.3) and significantly higher than rates for Whites (7.0%, 95% CI 5.8–8.4). SCI and dementia prevalence rates were highest in the lowest education category (SCI: 23.4%, 95% CI 19.1–28.4 vs dementia: 17.9%, 95% CI 14.4–22.0).

### 3.2 Agreement and strength of agreement of SCI with the probable dementia measure

The percent agreement of SCI with dementia status was 90.0%, with a PABAK statistic of 0.80 (95% CI 0.77–0.83; Table 2) interpreted as substantial strength of agreement (eTable S4).^43^ Across subgroups, the observed percent agreement between measures was higher in the younger and higher education subgroups and lower in the older and lower education subgroups, but did not differ significantly by sex or race and ethnicity (Table 2). In contrast, the kappa statistic was higher in the older age group (κ 0.51, 95% CI 0.44–0.59) and lowest in the youngest age group (κ 0.33, 95% CI 0.18–0.48), but did not differ significantly across educational categories (Table 2). This incongruence between the estimated percent agreement and the kappa statistics was largely driven by the imbalance in the marginal totals within age and educational subgroups (Figure 1 and Table 2). Indeed, the PABAK ranged from 0.87 (95% CI 0.84–0.91; interpreted as almost perfect agreement)^43^ in the youngest age group to 0.67 (95% CI 0.60–0.73; interpreted as substantial agreement)^43^ in the oldest age group, consistent with the patterns in the observed percent agreement across age categories. Similarly, the PABAK ranged from 0.63 (95% CI 0.56–0.70; interpreted as substantial agreement)^43^ in the lowest educational attainment category (those with less than a high school degree) to 0.88 (95% CI 0.83–0.92; interpreted as almost perfect agreement)^43^ in the highest educational attainment category (college graduate and higher).

**TABLE 2:** Percent agreement and strength of agreement between SCI and NHATS probable dementia measures, overall, and by age group, sex, race and ethnicity, and education.

	Percent agreement, %		Indices, %		Agreement strength, Est. (95% CI)	
Characteristics	Observed ($p_{o}$)^a^	Expected ($p_{e}$) ^a^	Prevalence (PI) ^b^	Bias (BI) ^b^	Kappa (κ)^c^	PABAK ($\kappa^{*}$)^c^
**Overall**	90.04	81.38	‐79.31	3.79	0.46 (0.40–0.53)	0.80 (0.77–0.83)
**Age group (y)**						
65–74	93.59	90.43	‐89.99	3.49	0.33 (0.18–0.48)	0.87 (0.84–0.91)
75–84	88.06	77.59	‐74.41	4.50	0.47 (0.38–0.56)	0.76 (0.72–0.81)
≥85	83.33	66.06	‐56.76	3.07	0.51 (0.41–0.60)	0.67 (0.60–0.73)
**Sex**						
Male	90.23	84.38	‐82.99	3.44	0.37 (0.27–0.48)	0.80 (0.76–0.85)
Female	89.89	79.18	‐76.50	4.05	0.51 (0.44–0.59)	0.80 (0.76–0.83)
**Race and ethnicity**						
White, non‐Hispanic	90.81	83.30	‐81.72	4.27	0.45 (0.37–0.53)	0.82 (0.79–0.85)
Black, non‐Hispanic	86.35	74.40	‐70.00	4.55	0.47 (0.35–0.59)	0.73 (0.66–0.79)
Hispanic	86.12	70.76	‐64.57	3.97	0.53 (0.32–0.73)	0.72 (0.59–0.85)
**Education**						
Less than high school	81.42	67.06	‐58.67	5.57	0.44 (0.34–0.53)	0.63 (0.5–0.70)
High school graduate	91.25	81.82	‐79.86	3.64	0.52 (0.41–0.63)	0.83 (0.78–0.87)
Some college	91.74	85.82	‐84.74	4.19	0.42 (0.24–0.59)	0.83 (0.78–0.89)
College graduate and higher	93.93	91.31	‐90.92	2.30	0.30 (0.11–0.49)	0.88 (0.83–0.92)

*Note*: Sample included *n* = 1936 observations (weighted *n* = 35,489,497). Other race and ethnicity category is not reported due to the small sample size (unweighted *n* = 73). All estimates are adjusted using round 2 analytic weights to produce a nationally representative sample and account for differential probabilities of selection and nonresponse in NHATS and were further adjusted to reflect that the analytic sample is a random one‐third sample of NHATS participants in 2012.

Abbreviations: CI, confidence interval; NHATS, National Health and Aging Trends Study; PABAK, prevalence‐and bias‐adjusted kappa.; SCI, subjective cognitive impairment; y, years.

^a^ Percent observed agreement ($p_{o}$) is defined as how much agreement is actually present or “observed” and is calculated as $p_{o}=\;\left(a+d\right)/N$ where a and d represent the frequencies in which the two instruments agree (a is when both instruments say “Yes” and denotes the true‐positive cases, TP; d is when both instruments say “No” and denotes the true‐negative cases, TN) and N is the total frequency of observation (eTable S3). Percent expected agreement ($p_{e}$) is the agreement that is expected to be present by chance alone and is calculated as $p_{e}=\left(\left(\left(a+b)\times (a+c\right))+(\left(c+d)\times (b+d\right)\right)\right)\;/N^{2}$ where a and d are defined as before in note ^¶^; b and c represent represent the frequencies in which the two instruments do not agree (b denotes the false‐positive cases, FP; c denotes the false‐negative cases, FN) and N is the total frequency of observation.

^b^ The prevalence index (PI) is the difference between the prevalence of TP and TN ratings. It is calculated as PI=(a−d)/N. A positive value of the index indicates that the prevalence of TP ratings exceeds that of TN ratings; a negative signage indicates the opposite relationship. The bias index (BI) represents the difference between the prevalence of FP and FN ratings. It is calculated as BI=(b−c)/N. A positive sign indicates that the prevalence of FP ratings exceeds that of FN ratings, with a negative signage indicating the opposite relationship.

^c^ Kappa (κ) denotes the Cohen and Conger's kappa and is calculated as $\kappa \;=\;\left(p_{o}-p_{e}\right)/\left(1-p_{e}\right)$. PABAK ($\kappa^{*}$) represents the Brennan and Prediger coefficient and is calculated as $\kappa^{*}=\;2p_{o}-1$. The extent of agreement is interpreted from benchmark scales in Landis and Kock (1977) represented in eTable S4.

Results from sensitivity analyses with a less‐restrictive NHATS definition of possible or probable dementia (eTable S7) were qualitatively similar to those based on the restrictive measure of probable dementia only (Table 2), although levels of percent agreement and measures of agreement strength were lower. For example, agreement rates and the PABAK statistics were lower in older and less‐educated individuals (eTable S7).

### 3.3 Predictors of disagreement between the SCI and probable dementia measures

Results from the logistic regression and log‐binomial models (Table 3) and from the Cox's proportional hazard and modified Poisson models (eTable S8) are strikingly similar in magnitudes. We focus on the log‐binomial models. The results indicate that the baseline relative risk (RR) of overall and false‐positive misclassification–disagreement between the SCI and probable dementia measures–were 0.12 (95% CI 0.07–0.18) and 0.10 (95% CI 0.06–0.17), respectively. Relative to older adults in the youngest age group, those in older age groups had significantly higher risk of overall disagreement (75–84 years: RR 1.72, 95% CI 1.26–2.36 and ≥85 years: OR 2.37, 95% CI 1.60–3.51) and false‐positive disagreement (75–84 years: RR 1.64, 95% CI 1.10–2.44 and ≥85 years: OR 2.17, 95% CI 1.30–3.64) between measures. There was no statistically significant difference in the risks of overall and false‐positive disagreement across sex and racial and ethnic populations. Relative to those with less than a high school education, having a higher education was associated with lower risks of overall and false‐positive disagreement. Only female sex had a statistically significant association with the risks of false‐negative disagreement (0.64, 95% CI 0.40–1.02). With the more liberal definition of dementia, misclassification rates varied by sex and race and ethnicity: rates were higher among males as well as non‐Hispanic Blacks and Hispanics (eTable S9 and eTable S10).

**TABLE 3:** Predictors of overall, false‐positive, and false‐negative misclassifications of probable dementia by SCI in logistic regression and log‐binomial models.

Predictors of misclassification	Logistic regression models						Log‐binomial models					
	Overall misclassification		False‐positive misclassification		False‐negative misclassification		Overall misclassification		False‐positive misclassification		False‐negative misclassification	
	OR	SE	OR	SE	OR	SE	RR	SE	RR	SE	RR	SE
**Intercept**	0.140^a^	(0.0347)	0.120^a^	(0.0341)	0.996	(0.629)	0.117^a^	(0.026)	0.103^a^	(0.027)	0.646	(0.306)
**Age group, y (ref = 65**–**74 y)**												
75–84	1.868^a^	(0.315)	1.754^a^	(0.368)	0.758	(0.420)	1.725^a^	(0.271)	1.642^b^	(0.325)	0.637	(0.266)
≥85	2.747^a^	(0.616)	2.439^a^	(0.689)	0.711	(0.386)	2.371^a^	(0.465)	2.172^a^	(0.558)	0.601	(0.268)
**Female (ref = Male)**	0.893	(0.158)	0.982	(0.199)	0.544^c^	(0.183)	0.917	(0.139)	0.987	(0.177)	0.641^c^	(0.148)
**Race and ethnicity (ref = White, non‐Hispanic)**												
Black, non‐Hispanic	1.266	(0.249)	1.184	(0.267)	0.934	(0.429)	1.227	(0.195)	1.178	(0.233)	0.910	(0.293)
Hispanic	1.096	(0.316)	1.002	(0.347)	0.829	(0.482)	1.103	(0.260)	1.021	(0.299)	0.745	(0.355)
**Education (ref = Less than high school)**												
High school graduate	0.467^a^	(0.109)	0.427^a^	(0.112)	0.939	(0.379)	0.537^a^	(0.108)	0.484^a^	(0.112)	1.056	(0.328)
Some college	0.420^a^	(0.132)	0.405^b^	(0.150)	1.030	(0.641)	0.482^a^	(0.130)	0.457^b^	(0.147)	1.015	(0.414)
College graduate and higher	0.333^a^	(0.087)	0.287^a^	(0.096)	1.741	(1.108)	0.392^a^	(0.092)	0.333^a^	(0.101)	1.548	(0.564)
**Observations**												
Unweighted	1863		1660		203		1863		1660		203	
Weighted	34,076,757		31,325,138		2,751,618		34,076,757		31,325,138		2,751,618	
**Number of strata**	56		56		52		56		56		52	
**Number of PSUs**	112		112		104		112		112		104	
**Degrees of freedom**	49		49		45		49		49		45	
**Adjusted Wald test F‐statistic**	9.20^a^		6.14^a^		0.76		–		–		–	

*Note*: Estimates are from logistic regression and log‐binomial models. Sample included *n* = 1863 observations (weighted *n* = 34,076,757). Other race and ethnicity category is not reported due to the small sample size (unweighted *n* = 73). All estimates are adjusted using round 2 analytic weights, to produce a nationally representative sample and account for differential probabilities of selection and nonresponse in NHATS and were further adjusted to reflect that the analytic sample is a random one‐third sample of NHATS participants in 2012. SE in parentheses; statistical significance:

Abbreviations: NHATS, National Health and Aging Trends Study; OR, odds ratio; RR, relative risk; SCI, subjective cognitive impairment; SE, standard error.

^a^ *p* < 0.01,

^b^ *p* < 0.05,

^c^ *p* < 0.1.

### 3.4 Sensitivity and specificity of SCI against probable dementia

Table 4 summarizes measures of the accuracy of SCI against our measure of probable dementia, by individual characteristics. SCI's sensitivity for detecting dementia was 63.5% overall, and highest among individuals aged ≥84 years (66.2%), females (68.8%), Hispanics (68.5%), and high school graduates (69.1%). Sensitivity was lowest among those in the youngest age group (55.3%), males (53.3%), and those in the highest education category (44.4%).

**TABLE 4:** Sensitivity and specificity of SCI against the NHATS probable dementia definition, by age group, sex, race and ethnicity, and education.

Characteristic	Sensitivity ^a^ (95% CI)	Specificity ^b^ (95% CI)
**Overall**	63.45 (63.40–63.51)	92.49 (92.48–92.50)
**Age group (y)**		
65–74	55.29 (55.16–55.42)	94.88 (94.87–94.89)
75–84	64.70 (64.62–64.79)	90.81 (90.80–90.83)
≥85	66.15 (66.06–66.23)	87.65 (87.61–87.68)
**Sex**		
Male	53.34 (53.24–53.43)	92.91 (92.90–92.93)
Female	68.84 (68.77–68.90)	92.16 (92.15–92.17)
**Race and ethnicity**		
White, non‐Hispanic	64.85 (64.78–64.91)	92.76 (92.75–92.77)
Black, non‐Hispanic	64.24 (64.09–64.40)	89.58 (89.54–89.61)
Hispanic	68.52 (68.37–68.67)	89.41 (89.36–89.45)
**Education**		
Less than high school	63.62 (63.54–63.70)	85.30 (85.27–85.33)
High school graduate	69.07 (68.98–69.16)	93.25 (93.23–93.26)
Some college	63.22 (63.06–63.38)	93.41 (93.39–93.43)
College graduate and higher	44.40 (44.22–44.57)	95.67 (95.65–95.68)

*Note*: Sample includes *n *= 1936 observations (weighted *n* = 35,489,497). All estimates are adjusted using round 2 analytic weights, to produce a nationally representative sample and account for differential probabilities of selection and nonresponse in NHATS and were further adjusted to reflect that the analytic sample is a random one‐third sample of NHATS participants in 2012. We assume the NHATS probable dementia definition to be the reference standard for dementia identification, as it is widely used in the literature to quantify dementia prevalence. We then assess the performance of the SCI measure against this NHATS probable dementia definition to assess the validity of SCI as a population‐based dementia risk identification tool. Other race and ethnicity category is not reported due to the small sample size (unweighted *n* = 73).

Abbreviations: CI, confidence interval; NHATS, National Health and Aging Trends Study; SCI, subjective cognitive impairment; y, years.

^a^ Sensitivity is the probability that the classifier produces a positive result in individuals with the condition of interest.

^b^ Specificity is the probability that the classifier produces a negative result in individuals without the condition of interest.

Specificity was 92.5% overall, highest among the youngest age group (94.9%), males (92.9%), non‐Hispanic Whites (92.8%), and those in the highest education category (95.7%), and lowest in the oldest age group (88.7%), females (92.2%), Hispanics (89.4%), and those in the lowest education category (85.3%).

Robustness analyses indicated that SCI had low sensitivity (33.4%) but high specificity (94.9%) against the NHATS possible or probable dementia definition (eTable S11). Sensitivity was highest in older and less‐educated individuals, females, and non‐Hispanic Blacks. In contrast, specificity was highest in younger and more educated individuals and non‐Hispanics (eTable S11).

## 4 DISCUSSION

We drew on national survey data to comparatively assess the prevalence of SCI and a validated measure of probable dementia, as well as the extent to which the degree of agreement between the two measures varies across individual characteristics. Consistent with prior literature, both SCI and probable dementia measures were highest among older age groups, Blacks and Hispanics, and individuals with lower educational attainment. However, contrary to prior studies,^58^, ^59^ we observed no significant sex differences in prevalence across measures. This may be due to our exclusion from the sample of older adults residing in a nursing home in rounds 1 or 2 of the NHATS.

As also expected, SCI prevalence was nearly 1.5 times that for probable dementia (12.2% vs 8.5%), given that not all cognitive impairments are dementia related. When we used a less‐restrictive measure of dementia risk, which includes individuals classified as having probable or possible dementia, the estimated risk of dementia nearly tripled to 25.1% (eTable S6). However, within subgroups, SCI prevalence was significantly higher than that for probable dementia, only for the younger age group, females, Whites, and persons with a college or higher degree.

The percent agreement between SCI and the validated measure of dementia was 90.0%, overall, with a PABAK statistic indicative of substantial strength of agreement ($\kappa^{*}$, 0.80). Overall and false‐positive misclassification (disagreement) rates varied across age groups and education categories: older adults in the older age groups had overall and false‐positive disagreement rates, whereas higher education correlated with lower disagreement rates. Females had lower false‐negative disagreement rates. Race and ethnicity did not significantly impact disagreement rates. The sensitivity and specificity of SCI against the validated probable dementia measure were 63.5% and 92.5%, respectively (Table 4), and comparable to those for the NHATS probable dementia definition against the “gold‐standard” ADAMS‐based criteria for dementia (sensitivity, 65.7%) and normal plus cognitive impairment with no dementia (CIND; specificity, 87.2%). SCI distinguished well between older adults with and without probable dementia, with PPV and NPV of 43.8% and 96.5%, respectively (eTable S12 and eFigure 1), and an AUC of 0.78 (eTable S13 and eFigure S2).

Overall, the results of this study supported our hypotheses, suggest that the SCI and probable dementia measures track each other well, and provide new support for the discriminatory properties of SCI in detecting dementia risk. Thus, monitoring SCI in routinely conducted large population‐based surveys (e.g., ACS, BRFSS, NHIS, etc.), may be a potentially low‐cost and easily accessible dementia surveillance tool. In combination with the routinely collected rich sociodemographic, socioeconomic, health, geographic, and contextual information in these surveys, tracking SCI may generate critical information about dementia risk, and serve as a tool to help evaluate national and local efforts to address the burden of cognitive impairment and dementia. Ascertaining comprehensive data can significantly improve the ability of policymakers to develop appropriate and targeted interventions and policies, such as increasing public awareness about dementia, promoting early detection and diagnosis of dementia, and routinely including cognitive health in public health campaigns. Interventions may also involve increasing awareness of and participation of Medicare beneficiaries—particularly those with SCI–in the Medicare Annual Wellness Visit (or AWV), which already incorporates SCI as a part of routine screening^15^, ^16^, ^17^ but remains underutilized,^4^, ^60^ despite being associated with increased likelihood of new dementia diagnosis.^12^ Such actions could benefit early detection of both dementia and other cognition‐related conditions, to permit targeted and timely interventions.

With no cost‐effective biomarkers for dementia identification and the limited evidence regarding the tradeoffs between the risks and benefits of routine dementia screening,^1^, ^2^ dementia surveillance continues to rely primarily on administrative medical claims records and community‐based studies, with important timing lags in data availability and access, and concerns about their representativeness of the diversity of the U.S. population. Although these data remain important to our understanding of dementia, they may produce biased estimates of dementia burden and mask important variability in disease patterns, particularly across smaller population subgroups and geographies. Our results suggest that tracking SCI may be a useful approach for dementia surveillance, and for ensuring that we can use a more diverse range of studies that do not include a battery of cognitive function questions and tests, to better understand the risk, impact, and burden of dementia across communities and population subgroups. Although we found heterogeneity in agreement of SCI with probable dementia by age group and educational attainment, this may simply be reflective of potential biases in the NHATS probable dementia algorithm, and specifically, from differences in performance of the three criteria used by the algorithm across subgroups. First, the NHATS measure is potentially vulnerable to diagnosis bias, given previously documented disparities in dementia diagnosis among U.S. community‐dwelling older adults.^61^, ^62^ Second, cognitive tests often lack cultural sensitivity, potentially underestimating cognitive performance in certain subgroups.^63^ In addition, the AD8 algorithm, a key component of cognitive assessment in the NHATS algorithm, is known to exhibit a “ceiling effect,” making it less sensitive for subpopulations with higher educational level.^64^ Despite validation studies with small, less‐diverse samples suggesting that the AD8 is less susceptible to biases,^33^ evidence gaps persist. For instance, a study at Washington University School of Medicine showed comparable AD8 performance between racial and age groups, albeit with a small sample size (*n *= 325) and mostly Caucasians,^31^ highlighting the need for further research across diverse subgroups. Our finding of no association between misclassification rates and race and ethnicity suggests the utility of SCI for studying racial disparities in dementia risk. Third, potential differences in the accuracy of the TICS and TICSm by subpopulations may contribute to observed heterogeneity. For example, recent analyses have reported on variability across studies in the cutoffs for the TICS and TICSm for identifying MCI and dementia, with the thresholds being potentially impacted by education levels.^65^ This implies that the TICS and TICSm may vary in performance based on educational attainment, as highly educated individuals typically perform better in cognitive assessments, potentially resulting in less‐extreme cut‐off scores. In addition, Gianattasio et al. (2019) found that the accuracy of five commonly used algorithms (including items from the TICS) for dementia classification, varied across age groups, education level, race and ethnicity, and respondent status (self vs proxy).^66^ Further research is needed, therefore, to elucidate whether SCI is consistently assessed and performs comparably across subpopulations.

This study has several limitations. First, we only compared SCI to the NHATS probable dementia definition, a measure often criticized for its heavy focus on domains of language and memory, sensitivity to education level, and limited ability to differentiate CIND or MCI from dementia, due to differing definitions and evolving diagnostic criteria.^34^, ^67^, ^68^ Moreover, the NHATS dementia definitions neither involve biomarkers nor laboratory data, both of which could greatly improve dementia ascertainment. Despite these limitations, the NHATS measure was shown to be valid and reliable measures of dementia. Second, the SCI measure may not sufficiently discriminate between dementia‐related cognitive impairment and cognitive difficulties that mimic dementia but are potentially unrelated to it, including those resulting from a physical, mental, or emotional problem, such as trauma, depression, anxiety, mood disorders or insomnia, or from other reversible causes of cognitive impairment, such as urinary tract infection, vitamin deficiencies, and medications. In addition, SCI may capture MCI, or CIND, both of which are not well captured by the NHATS probable dementia definition. However, controlling for some of these factors improved SCI's accuracy in detecting dementia. Third, this study was cross‐sectional, and may not capture temporal changes in SCI status or relationships between SCI and dementia risk.

Notwithstanding these limitations, our analysis suggests that routinely fielded population‐based surveys such as ACS may be useful dementia surveillance tools, through the monitoring of SCI. Indeed, a recent ACS‐based study reported a 1.8 percentage point decline in SCI prevalence between 2008 and 2017,^69^ concomitant with reported declines in dementia prevalence of similar order of magnitude,^70^ and underscoring the public health value of tracking SCI for the purposes of dementia surveillance. States are become increasingly invested in understanding the burden and fiscal impacts of cognitive impairment and dementia and addressing disparities in dementia. As such, it is paramount to validate SCI and similar subjectively reported measures of cognitive impairment against established validated measures of dementia and demonstrating that these measures are consistently assessed and perform comparably across subpopulations.

## CONFLICT OF INTEREST STATEMENT

None.

## CONSENT STATEMENT

All human subjects participating in the National Health and Aging Trends Study (NHATS) provided written informed consent. A separate consent was, therefore, not necessary for this study. NHATS was approved by the Johns Hopkins Bloomberg School of Public Health Institutional Review Board (IRB) and these analyses were deemed exempt from review.

## Supporting information

Supporting information

Supporting information
