# Supplementary material for: Analysis of agreement between measures of subjective cognitive impairment and probable dementia in the National Health and Aging Trends Study
Source: Alzheimers Dement. 2024 Mar 1;20(4):2817–29. doi: 10.1002/alz.13758 (PMC11032562; doi:10.1002/alz.13758)
Supplement: Supplementary file 1 — Supporting information [file ALZ-20-2817-s001.docx]

**SUPPLEMENTARY MATERIALS**

**Analysis of Agreement Between Measures of Subjective Cognitive Impairment and Dementia in the National Health and Aging Trends Study**

Linda C. Chyr, MPH, PhD^1^, Jennifer L. Wolff, PhD^2^, Julie Zissimopoulos, PhD^3,4^, Emmanuel F. Drabo, MPhil, PhD^2^

^1^ Enterprise Analytics Core, Elevance Health, Inc., Wilmington, DE 19801

^2^ Department of Health Policy and Management, John Hopkins Bloomberg School of Public Health, Baltimore, MD 21205

^3^ Sol Price School of Public Policy, University of Southern California, Los Angeles, CA 90089-3333

^4^ Leonard D. Schaeffer Center for Health Policy, University of Southern California, Los Angeles, CA 90089-3333’

1. **Introduction**

Prior literature suggest that subjectively reported cognitive impairment and cognitive difficulty may be warning signs of dementia, since difficulty with and decline in cognition are hallmark characteristics of dementia. Large population-based surveys routinely conducted in the United States already collect information on subjective cognitive impairment and cognitive decline. This could potentially permit a population-based surveillance of dementia risk and inform screening strategies to detect early dementia onset. To do this requires, however, establishing the validity of these subjectively reported difficulties with cognition against standard reference measures of dementia. This study aims to fill this evidence gap. We leverage data from the National Health and Aging Trends Study (NHATS) to assess the performance of the subjective cognitive impairment (SCI) measure against a validated measure of probable dementia.

The SCI measure can be constructed from responses to the Cognitive Disability item of the U.S. Census Bureau’s six-items Disability Questionnaire, as further described in subsequent sections. In NHATS, the Cognitive Disability item was fielded in Round 2 (2012) among a random one third sample of NHATS participants. Because SCI and dementia were concurrently assessed among the same subset of NHATS participants during that round, we can leverage that information to assess the performance of SCI as a dementia surveillance tool, and to screen for individuals that might be in greater need for more resource-intensive dementia assessment, such as in clinical settings.

In this supplementary appendix, we provide more details on the technical concepts and statistics used throughout the manuscript, and how they are measured. We also provide additional results in support of the key findings presented in the manuscript, which we could not include in the main text. This supplementary appendix is therefore organized as follows: First, we provide an overview of the cognitive disability item of the U.S. Census Bureau’s 6-items Disability Questionnaire. We discuss how this item is operationalized across different surveys and how prevalence estimates of SCI vary across three major surveys routinely used to inform disability-related policies in the U.S. Next, we provide technical details on important statistics used in the main article to assess the accuracy of the SCI measure against the validated measure of probable dementia. Finally, we provide additional analyses and results that support the key findings from the study.

1. **NHATS dementia definitions**

Probable dementia is defined by the presence of any of the following: 1) a self- or proxy-report of a dementia diagnosis, as defined above; 2) a proxy-reported AD8 score ≥ 2; ^1^ or 3) a score of 1.5 standard deviations (SDs) or less below the mean on at least two of three of the cognitive functioning domains assessed by the TICSm and Clock-Drawing Test.^2^ Possible dementia is defined by a score of 1.5 SDs or less below the mean on at least one of the three cognitive functioning domains. Participants who meet none of the criteria for probable or possible dementia are classified as having no dementia.

1. **Subjectively reported measures of cognitive impairment**
   1. **Subjective cognitive impairment (SCI)**

Subjective cognitive impairment is assessed through the Cognitive Disability item of the U.S. Census Bureau’s Disability Questionnaire. Versions of the Census Bureau’s Disability Questionnaires have been fielded in many major population-based surveys. Below, we describe the particularities of alternative versions of the cognitive disability item. eTable 1 summarizes potential similarities and differences in the prevalence of outcomes based on these measures for year 2018, the latest year for which comprehensive data are available across the selected surveys examined.

- 1. **Brief overview of other selected surveys permitting measurement of SCI**
     1. **The American Community Survey (ACS)**

The American Community Survey (ACS) is a large annual nationwide survey of approximately 3.5 million randomly sampled U.S. household units, designed to supplement the decennial census, and additionally collects information on the economic, social, housing, and demographic characteristics of communities in all states and the District of Columbia (DC) on a continuous basis. The ACS has included the disability questionnaire since 1999 as part of its core module.

- - 1. **The Behavioral Risk Factors Surveillance Survey (BRFSS)**

The Behavioral Risk Factors Surveillance Survey (BRFSS) is an annual health behavior survey administered in every state, DC, and the U.S. territories. In BRFSS, data are collected monthly and independently by each state, so methodologies may vary, and pooled state-level variability may be reflective of such methodological differences. The cognitive disability item is included in Cognitive Decline Module of BRFSS, which was offered for the first time in 2011, as the Cognitive Impairment Module, and then subsequently included as an official optional module between 2011 and 2013 for 47 states and territories. In 2015, the Cognitive Decline Module was added as an official optional module for all states. Overall, 49 states (all except Pennsylvania), Puerto Rico, and DC administered this revised module between 2015 and 2016. The module was administered in Pennsylvania for the first time in 2018.^3,4^

- - 1. **The National Health Interview Survey (NHIS)**

The National Health Interview Survey (NHIS) is a cross-sectional household interview survey conducted annually since 1997 by the National Center for Health Statistics (NCHS), within the Centers for Disease Control and Prevention (CDC), and is nationally representative of the US civilian noninstitutionalized population’s health status, health care access, and progress toward achieving national health objectives.^5,6^

In NHIS, the cognitive disability item is included in the Adult Functioning and Disability (NHIS-AFD) supplement, fielded with the Sample Adult module since 2010. Prior to 2010, this question was included in the core of the NIHS survey instrument. During subsequent surveys, the questions were only administered to a subset (a half or a quarter) of the adult sample. In 2018, the NHIS-AFD was incorporated into the Sample Adult File and administered to all adult respondents (≥ 18 years).^7^

| **eTable 1: Definitions of measures of SCI in the ACS, BRFSS, and NHIS** | | | |
| --- | --- | --- | --- |
| **Characteristics** | **Surveys** | | |
|  | **ACS** | **BRFSS** | **NHIS** |
| Cognitive item of the U.S. Census Bureau’s Disability Questionnaire | “Because of a physical, mental, or emotional problem, does this person have difficulty remembering, concentrating, or making decisions?” | “Because of a physical, mental, or emotional condition, do you have serious difficulty concentrating, remembering, or making decisions?” | |
| State coverage | All states + DC | 2018: 6 states  2017: 9 states  2016: 21 states  2015: 34 states | All states + DC |
| Universe | Civilian non-institutionalized population residing in the U.S., and aged 18 years and older. | Civilian non-institutionalized population residing in the U.S., and aged 18 years and older. | Adults 18 years and older who were asked the family disability questions, were randomly selected to receive the Functioning and Disability section |
| Years analyzed | 2015-2018 | 2015-2018 | 2015-2018 |
| Sample size (≥ 65y), n | 2,433,119 | 151,870 | 31,903 |
| SCI prevalence, % (SE) | 10.0 (0.026) | 8.9 (0.191) | 8.6 (0.281) |
| **Abbreviations:** SCI, subjective cognitive impairment; ACS, American Community Survey; BRFSS, Behavioral Risk Factor Surveillance System; NHIS, National Health Interview Survey; DC, District of Columbia; y, year; SE, standard error.  **Notes:** Some have referred to this measure as “serious cognitive difficulty”.^8^ To be consistent with the BRFSS nomenclature, we refer to the ACS measure as subjective cognitive impairment (SCI). | | | |

- - 1. **Other surveys**

Other routinely fielded population-based cross-sectional and longitudinal surveys exist and include measures of cognitive impairment. Their exhaustive overview is beyond our scope. They include, for example: the Current Population Survey Annual Social and Economic Supplement (CPS-ASEC), the National Health and Nutrition Examination Survey (NHANES), the Panel Study of Income Dynamics (PSID), the Medical Expenditure Panel Survey (MEPS), the Survey of Income and Program Participation (SIPP), and the American Housing Survey (AHS).

- 1. **Other subjectively reported measures of cognition**

Some surveys collect additional data on cognition. Given that these measures are not included in the NHATS, we cannot directly evaluate their accuracy against the NHATS validated measure of probable dementia. In ongoing work, we are developing indirect validations of these measures for the purposes of dementia surveillance. For example, the Cognitive Decline Module and Caregiver Module of BRFSS permit measurement of subjective cognitive decline (SCD), through a question which asks respondents aged 45 years and older the following: *“During the past 12 months, have you experienced confusion or memory loss that is happening more often or is getting worse?”* ^9^ NHIS has a measure of cognitive impairment due to senility.

1. **Definitions of covariates**

We categorized age in three groups (i.e., 65-74, 75-84, and ≥85 years). Sex was defined as the respondent’s reported biological sex (i.e., male, female). Race and ethnicity were operationalized using the NHATS race variable, and respondents were classified as non-Hispanic white (White henceforth), non-Hispanic black (Black henceforth), Hispanic, and other race and ethnicity (Other henceforth), which included respondent of American Indian, Asian, native Hawaiian and Pacific Islander race and ethnicity. We constructed educational attainment as a 4-level categorical variable: 1) having less than a high school education, 2) being a high school graduate, 3) having some college education, and 4) having a bachelor’s degree or higher.

| **eTable 2: Characteristics of the NHATS respondents who participated in the Disability Questionnaire, by response type to the Cognitive Disability item** | | | | | | |
| --- | --- | --- | --- | --- | --- | --- |
|  |  |  | **SCI Status** | | |  |
| **Characteristics** | **Total** |  | **No SCI** | **SCI** | **Missing** | **p-value^‡^** |
| **Overall, N (%)** |  |  |  |  |  |  |
| Unweighted | 1,954 |  | 1,647 | 289 | 18 | <0.001 |
| Weighted | 35,804,808 |  | 31,146,614 | 4,342,883 | 315,311 | <0.001 |
| **Dementia status^†^** |  |  |  |  |  | <0.001 |
| No dementia | 1,331 (74.7) |  | 1,248 (81.0) | 74 (31.4) | 9 (48.0) |  |
| Possible dementia | 221 (8.6) |  | 79 (3.5) | 138 (43.8) | 4 (25.1) |  |
| Probable dementia | 402 (16.7) |  | 320 (15.5) | 77 (24.7) | 5 (26.9) |  |
| **Age (y), mean (SD)** | 76.2 (7.2) |  | 75.6 (6.8) | 79.8 (8.6) | 77.2 (6.9) | <0.001 |
| **Age group (y)** |  |  |  |  |  | <0.001 |
| 65-74 | 701 (48.8) |  | 643 (52.0) | 53 (27.0) | 5 (34.0) |  |
| 75-84 | 784 (35.7) |  | 654 (34.5) | 123 (43.8) | 7 (45.2) |  |
| ≥85 | 469 (15.5) |  | 350 (13.5) | 113 (29.2) | 6 (20.8) |  |
| **Female** | 1,144 (56.8) |  | 945 (55.7) | 187 (63.9) | 12 (58.8) | 0.086 |
| **Race and ethnicity** |  |  |  |  |  | 0.027 |
| White, non-Hispanic | 1,355 (81.5) |  | 1,163 (82.3) | 178 (75.0) | 14 (85.2) |  |
| Black, non-Hispanic | 411 (8.0) |  | 330 (7.6) | 78 (11.4) | 3 (5.3) |  |
| Hispanic | 114 (6.5) |  | 90 (6.0) | 24 (10.5) | 0 (0.0) |  |
| Other, non-Hispanic | 74 (4.0) |  | 64 (4.1) | 9 (3.1) | 1 (9.5) |  |
| **Education** |  |  |  |  |  | <0.001 |
| Less than high school | 502 (20.4) |  | 368 (17.8) | 128 (39.0) | 6 (28.1) |  |
| High school graduate | 663 (35.4) |  | 566 (35.6) | 92 (34.4) | 5 (28.5) |  |
| Some college | 341 (18.4) |  | 301 (18.8) | 35 (14.5) | 5 (33.6) |  |
| College graduate and higher | 448 (25.8) |  | 412 (27.9) | 34 (12.1) | 2 (9.8) |  |
| **Residential care not nursing home, n (%)** | 116 (5.6) |  | 82 (4.4) | 34 (14.2) | 0 (0.0) | <0.001 |
| **Proxy respondent, n (%)** | 132 (5.0) |  | 38 (2.0) | 93 (26.6) | 1 (1.3) | <0.001 |
| **Abbreviations:** NHATS, National Health and Aging Trends Study; SCI, subjective cognitive impairment; SD, standard deviation; y, years.  **Notes:** The analytic sample is a random one-third sample of NHATS participants in 2012 who did not reside in a nursing home facility in 2011 and 2012 and participated in the Disability Questionnaire. Data are presented as mean (SD) for continuous measures, and % for categorical measures. Percentages are of column total and are unweighted.  ^†^ Dementia refers to the NHATS probable dementia definition; no dementia includes NHATS categorization of possible dementia.  ^‡^ Determined by Pearson’s chi-square test for binary and categorical variables and student’s t-test for continuous variables. | | | | | | |

1. **Agreement between measures**

We defined agreement between the SCI measure and the NHATS probable dementia definition as situations where respondents were classified as having both SCI and dementia or as having neither SCI nor dementia (i.e., $a + d$ in eTable 3); all other situations are defined as cases of disagreement between SCI and the dementia definition (i.e., $b + c$ in eTable 3). We then constructed a dummy variable taking the value 1 to denote agreement, and 0 otherwise. We then used this new variable to calculate the percent agreement between the SCI measure and dementia, defined as the proportion of respondents with an observed agreement between the SCI and dementia status.

| **eTable 3: Definition of agreement and disagreement between SCI and the NHATS probable dementia definition and derivation of sensitivity, specificity, and predictive values statistics** | | | | |
| --- | --- | --- | --- | --- |
| **Disability Questionnaire’s Measure of SCI** | | **Has Dementia**  **(NHATS Probable Dementia Definition^†^)** | | **Metrics** |
|  |  | **Yes** | **No** |  |
| **Has SCI** | **Yes** | a^*^  True positive | b^¶^  False positive | PPV = [a/(a + b)] |
|  | **No** | c^¶^  False negative | d^*^  True negative | NPV = [d/(c + d)] |
| **Metrics** | | Sensitivity = [a/(a + c)] | Specificity = [d/(b + d)] |  |
| **Abbreviations:** SCI, subjective cognitive impairment; NHATS, National Health and Aging Trends Study; PPV, positive predictive value; NPV, negative predictive value.  **Notes:**  ^*^ Agreement between measures  ^¶^ Disagreement between measures  † We assume that the NHATS probable dementia definition to be the reference standard for dementia identification, as it is widely used in the literature to quantify dementia prevalence. We then assess the performance of the SCI measure against this NHATS probable dementia definition to assess the validity of SCI as a population-based dementia risk identification tool. | | | | |

- 1. **Measure of the strength of agreement – the Cohen-Conger’s kappa statistic and the Brennan-Prediger’s prevalence-adjusted, bias-adjusted kappa (PABAK)**

To assess the strength of the observed agreement between the SCI measure and the validated dementia measure, we calculated the Cohen-Conger’s kappa statistic ($\kappa$) and the Brennan-Prediger’s prevalence-adjusted, bias-adjusted kappa (PABAK, $\kappa^{*}$).

- - 1. **Cohen-Conger’s kappa statistic**

The Cohen’s kappa statistic, $\kappa$, is the difference between the observed and expected agreement between measures, $(p_{o} - p_{e})$, normalized by their expected disagreement, ($1 - p_{e}$), and is expressed as:

| $\kappa= (p_{o} - p_{e})/(1 - p_{e})$, | (1) |
| --- | --- |

where:

| $p_{o} = 100\times(a+ d)/N$, | (2) |
| --- | --- |

| $p_{e} = 100\times([\left( a+ b \right)\times\left( a+c \right)]/N+[\left( c+d \right)\times\left( b+d \right)]/N)/N$, | (3) |
| --- | --- |

$N = a + b + c + d$, and $a$, $b$, $c$ and $d$ are defined as in eTable 3.

By adding and subtracting 1 from the numerator of the expression in Eq. (1), we can derive a different expression of kappa:

| $\kappa= 1- (1-p_{o})/(1 - p_{e})$ | (4) |
| --- | --- |

This reformulation of kappa can be interpreted as one minus the ratio of the observed disagreement to the expected disagreement between the two measures.

Practically, the Cohen-Conger’s kappa removes the possibility of the observed agreement between two measures is due to chance. Thus, $\kappa$ measures the number of correct predictions that a measure will make against a reference measure, that cannot be explained by a random guess, and corrects the evaluation bias by considering the correct classification by a random guess.

The Landis and Koch benchmark scale (eTable 4) is commonly used to interpret the value of kappa, which measures the strength of agreement between measures.^51,52^

| **eTable 4: Benchmark scales for strength of agreement for the Cohen-Conger’s kappa statistics^*^** | |
| --- | --- |
| **Kappa Statistic,** $\boldsymbol{\kappa}$ | **Interpretation** |
| <0.00 | Poor |
| 0.00-0.20 | Slight |
| 0.21-0.40 | Fair |
| 0.41-0.60 | Moderate |
| 0.61-0.80 | Good/Substantial |
| 0.81-1.00 | Very good/Almost Perfect |
| **Note:** *Landis and Koch (1977)^10^. | |

- - 1. **Brennan-Prediger’s PABAK statistic**

From Eq. (1) - (4), it can be noticed that the magnitude of the $\kappa$ is influenced by other factors such as disease prevalence, bias, and nonindependence of ratings. These influences may potentially complicate the interpretation of kappa provided above. The first two factors are more relevant for the present analysis as the assumption of independence of respondents and ratings satisfied.

Prevalence and bias effects can lead to well-known paradoxes first discussed by Feinstein and Cicchetti (1990).^49^ First, the chance correction process can yield a relatively high observed agreement value into a low value of $\kappa$ with a large percent expected agreement, due to the prevalence effect, so that for two different values of expected agreements, the resulting $\kappa$ values may grossly differ in magnitude for the same value of observed agreement. A prevalence effect exists when the proportion of agreements on the positive classification differs from that of the negative classification (i.e., difference between cells $a$ and $d$ in eTable 3), often expressed by the prevalence index:

| $PI=(a-d)/N$ | (5) |
| --- | --- |

With a high prevalence index (i.e., very high or very low prevalence of a positive rating), the chance agreement becomes also high, and kappa is reduced accordingly.

Second, due to the bias effect, “unbalanced marginal totals produce higher values of $\kappa$ than more balanced totals.” ^49^ A bias effect exists when there is disagreement between measures on the proportion of positive (or negative) cases (i.e., difference between cells $b$ and $c$ in eTable 3). This is often expressed by the bias index:

| $BI=(b-c)/N$ | (6) |
| --- | --- |

The value of $\kappa$ is higher in the presence of a large bias than when bias is low or nonexistent. Unlike prevalence, the effect of bias is also greater when $\kappa$ is small than when it is large.^50^

Because the magnitude of $\kappa$ is affected by both the prevalence of the condition and bias, $\kappa$ becomes difficult to interpret meaningfully on its own, without accounting for the prevalence and bias indices described in Eq. (5) and (6). The prevalence-adjusted, bias-adjusted kappa (PABAK, $\kappa^{*}$) can be used to account for these effects. PABAK adjusts the Cohen-Conger’s kappa statistic for both prevalence and bias,^50^ by replacing, in eTable 3, $b$ and $c$, and $a$ and $d$, by their respective averages, $m = (b + c)/2$, and $n = (a + d)/2$. Since $p_{e}=0.5$.

the resulting PABAK, is:

| $\kappa^{*}= 2p_{o}-1$, | (7) |
| --- | --- |

PABAK can be interpreted using the same benchmarks used to interpret the Cohen-Conger’s kappa (eTable 4).

Results from Table 2 of the main text suggest a percent agreement of SCI with dementia status of 90.0%, with a PABAK statistic of 0.80 (95% CI, 0.77-0.83; Table 2) interpreted as substantial strength of agreement (eTable 4).^43^ The reader will notice that this relationship is masked when one were to solely rely on the Cohen and Conger’s kappa value of 0.46 (95% CI, 0.40-0.53), interpreted as “moderate” agreement.^43^ In this case, the very high prevalence of true-negative responses in the population (i.e., individuals who are classified as having neither probable dementia nor SCI) compared to the prevalence of true-positive responses (i.e., individuals who are classified as having probable dementia and SCI) has resulted in a high negative prevalence index (-79.3%) and a low value of kappa, despite a 90% observed agreement between SCI and probable dementia ratings. The bias index is 3.79%, suggesting less pronounced imbalance in the prevalence of false-positive and false negative responses.

1. **Summary statistics for the SCI measure against the NHATS probable dementia definition**

Diagnostic accuracy is another way for assessing the degree of agreement between a classifier and a reference standard for the ability to identify a target condition. It is typically measured by examining a consecutive series of well-defined individuals participating in a study, who undergo both the classifier and reference tests in a blinded fashion. In our specific application, the classifier is SCI, and the target condition is dementia, as assessed by the NHATS probable dementia definition. We are interested in assessing the accuracy of SCI in identifying persons assessed as having dementia. The starting point is the construction of a 2 x 2 table with the results from the classifier on one side and those of the reference standard on the other (as illustrated above in eTable 3).

- 1. **Sensitivity, specificity of SCI against dementia**

The sensitivity of a classifier against a standard reference for a condition is the proportion of individuals assessed as having the condition by the standard measure (the NHATS probable dementia definition), that are also classified as having it according to the classifier (SCI). Mathematically, it is the ratio:

| $Sensitivity= TPR=a/(a+c),$ | (8) |
| --- | --- |

otherwise known as the true positive rate (TPR). It is also one minus the false negative rate ($FNR$), $c/(a + c)$ – since $c/(a + c) = [1 - a/(a + c)] = (1 - TPR)$– which represents the proportion of individuals with the condition that are classified as not having the condition.

Specificity represents the proportion of individuals with the condition that are classified as not having the condition. Mathematically, this is the ratio:

| $Specificity= TNR=d/(b+d),$ | (9) |
| --- | --- |

otherwise known as the true negative rate ($TNR$). It is also one minus the false positive rate ($FPR$), $b/(b + d)$ – since $b/(b + d) = [1 - d/(b + d)] = (1 - TNR)$ – which represents the proportion of individuals without the condition that are classified as having the condition. eTable 3 summarizes the derivation of these statistics for SCI (classifier) against the NHATS probable dementia definition (reference standard measure). Therefore, a classifier with high sensitivity will have high $TPR$ but a low $FNR$, and as such, can be used to rule out the presence of a condition. Similarly, a classifier with high specificity will have a low $FPR$, but high $TNR$, and as such, can be used to rule in a condition. The goal is therefore to choose a classifier with high sensitivity and specificity.

- 1. **Predictive values of SCI against dementia**

Diagnosis process is concerned with whether a person has a condition of interest rather than on sensitivity and specificity values of the classifier. Hence, the focus on the probability the condition being present in individuals classified as having it, and the probability of the condition being absent in individuals classified as not having it. These probabilities are known as post-test statistics (as opposed to sensitivity and specificity which are pre-test probabilities) and may be more informative in guiding at the implementation stage of the classifier. They include the positive and negative predictive values ($PPV$ and $NPV$).

Positive predictive value ($PPV$) represents the proportion of individuals classified as having the condition who have the condition (as assessed by the reference standard measure). Mathematically, this is ratio $a/(a + b)$, defined as the predictive value of positive classification. Negative predictive value ($NPV$) represents the proportion of individuals without the condition, as determined by both the classifier and the reference standard measure. Mathematically, this is the ratio $d/(c + d)$. eTable 3 summarizes the derivation of these statistics for SCI (classifier) against the NHATS probable dementia definition (reference standard measure). The $PPV$ is related to sensitivity, specificity, and prevalence through the following relationship:

| $PPV = \frac{(Sensitivity \times Prevalence)}{[\left( Sensitivity \times Prevalence \right)+ \left( 1 - Specificity \right)\times(1 - Prevalence)]}$ | (10) |
| --- | --- |

Similarly, $NPV$ is related to sensitivity, specificity, and prevalence as follows:

| $NPV = \frac{Specificity \times(1- Prevalence)}{[Specificity \times(1- Prevalence)+ \left( 1 - Sensitivity \right)\times Prevalence]}$ | (11) |
| --- | --- |

The results above suggest that classifiers with same levels of sensitivity and specificity may yield different $PPV$ and $NPV$ values in groups with different prior probabilities. For example, in our application, the $PPV$ of SCI against dementia varies greatly by the select sociodemographic and socioeconomic characteristics of age, sex, race and ethnicity and education (Table 4). The $PPV$ is lower in low-risk (younger age) groups and higher in high-risk (older age) groups, as dementia prevalence increases with older age. In contrast, the $NPV$ is higher in low prevalence age groups, and lower in high prevalence age groups. Indeed, there is a positive relationship between $PPV$ and prevalence, and a negative relationship between $NPV$ and prevalence.

Hence, while assessing the post-test accuracy of a classifier, we need to also consider the prior probability of the disease (i.e., prevalence) in the population of interest.

Therefore, for a given classifier, one cannot concomitantly increase sensitivity and specificity; increasing sensitivity comes at the expense of reducing specificity, and vice versa.

- 1. **Likelihood ratios**

The likelihood ratio ($LR$) is a measure of the performance of a classifier, which incorporates both sensitivity and specificity together. There are two different $LR$ statistics, including the positive and negative likelihood ratios.

*The positive likelihood ratio*$, LR(+)$*,* of a classifier is the ratio of the probability of obtaining positive result from the classifier in individuals with the condition to the probability of obtaining a positive result from the classifier in individuals without the condition:

| $LR(+) = \frac{Sensitivity}{(1 - Specificity)}$ | (12) |
| --- | --- |

The $LR(+)$ represents the likelihood that a classifier will produce a positive result in individuals with the condition of interest, relative to the same result in individuals without the condition. Like the odds ratio, the value of $LR(+)$ ranges from 0 to positive infinity. A $LR(+)$ value of 1 indicates that the likelihood of the presence of the condition is no more or less than chance. A value higher than 1 indicates that the classifier is more successful than chance at producing the positive results of the reference standard. Conversely, a value below 1 indicates that the classifier is worse than chance at producing the positive results of the reference standard. As the LR+ value becomes large, this is indicative of a higher discrimination capacity of the classifier, leading to greater change from the prior probability to the posterior disease probability (i.e., $PPV$). When the $LR(+)$ value is equal to 1, the $TPR$ is also equal to the FPR, thus indicating that the classifier is no more informative than chance. A $LR(+)$ value of 10 or higher is indicative of a good classifier for ruling in a condition.

*The negative likelihood ratio,* $LR(-)$*,* of a classifier is the ratio of the probability of obtaining a negative result from the classifier in individuals with the condition, to the probability of obtaining a negative result from the classifier in individuals without the condition. Its mathematical expression is as follows:

| $LR(-) = \frac{(1-Sensitivity)}{Specificity}$ | (13) |
| --- | --- |

As the value of the $LR(-)$ falls further below 1, the classifier becomes increasingly successful. Conversely, as the ratio nears 1, the classifier is unsuccessful. In general, a$LR(-)$value below 0.1 is indicative of a good classifier for ruling out a condition.

From the equations above, likelihood ratios ($LR(+)$ and $LR(-)$) do not depend on the prevalence of the condition in the population. This makes them useful measures for evaluating the performance of alternative classifiers against a reference standard and against each other, even for rare events. This contrasts with the other statistics such as the $PPV$ and $NPV$, which favor classifiers that always predict a negative outcome for rare events. In addition, LRs are a useful way for conveying the meaning and properties of a classifier or diagnostic test to clinicians and practitioners.^11^

- 1. **Relationship between posterior and prior probabilities**

As discussed above, there is a relationship between the prior probability (*i.e., disease prevalence*) and the posterior probabilities ($PPV$ *and* $NPV$) of a classifier. Specifically, $PPV$ is an increasing function of both the prior probability and the positive likelihood ratio ($LR(+)$), as seen in the following expression, also illustrated in Supplementary eFigure 1:

| $PPV = \frac{1}{1+\left( \frac{1}{LR(+)} \right)\times\left( \frac{1-Prevalence}{Prevalence} \right)}$ | (14) |
| --- | --- |

As illustrated in Supplementary eFigure 1, the $PPV$ curve moves closer to the upper left corner at any given level of the prior probability, as the value of $LR(+)$ increases. When the $LR(+)$ value equals to 1 (45-degree, diagonal line), the posterior probability is equal to the prior probability, so that the classifier is not informative in terms of ruling in the condition. However, as the curve nears the upper left corner of the figure, the classifier’s performance in ruling in a condition improves. This holds for any fixed level of the prior probability.

In contrast, $NPV$ is a decreasing function of both the prior probability and the negative likelihood ratio ($LR(-)$), as shown in the following expression, also illustrated in eFigure 1:

| $NPV = \frac{1}{1+\left( \frac{Prevalence}{1-Prevalence} \right)\times LR(-)}$ | (15) |
| --- | --- |

As Supplementary eFigure 1, shows, the $NPV$ curve moves closer to the bottom right corner at any given level of the prior probability, as the value of $LR(-)$ increases. At the 45-degree line, where the $LR(-)$ value equals to 1, the posterior probability is equal to the $NPV$. Hence, the classifier is not informative in terms of ruling out the condition. However, as the curve nears the upper left corner of the figure, the classifier’s performance in ruling in a condition improves. This holds also for any fixed level of the prior probability.

| **eFigure 1: Relationship between dementia prevalence and the predictive values of the SCI measure against a validated measure of dementia in NHATS (sensitivity, 63.45%; specificity, 92.49%)** |
| --- |
| 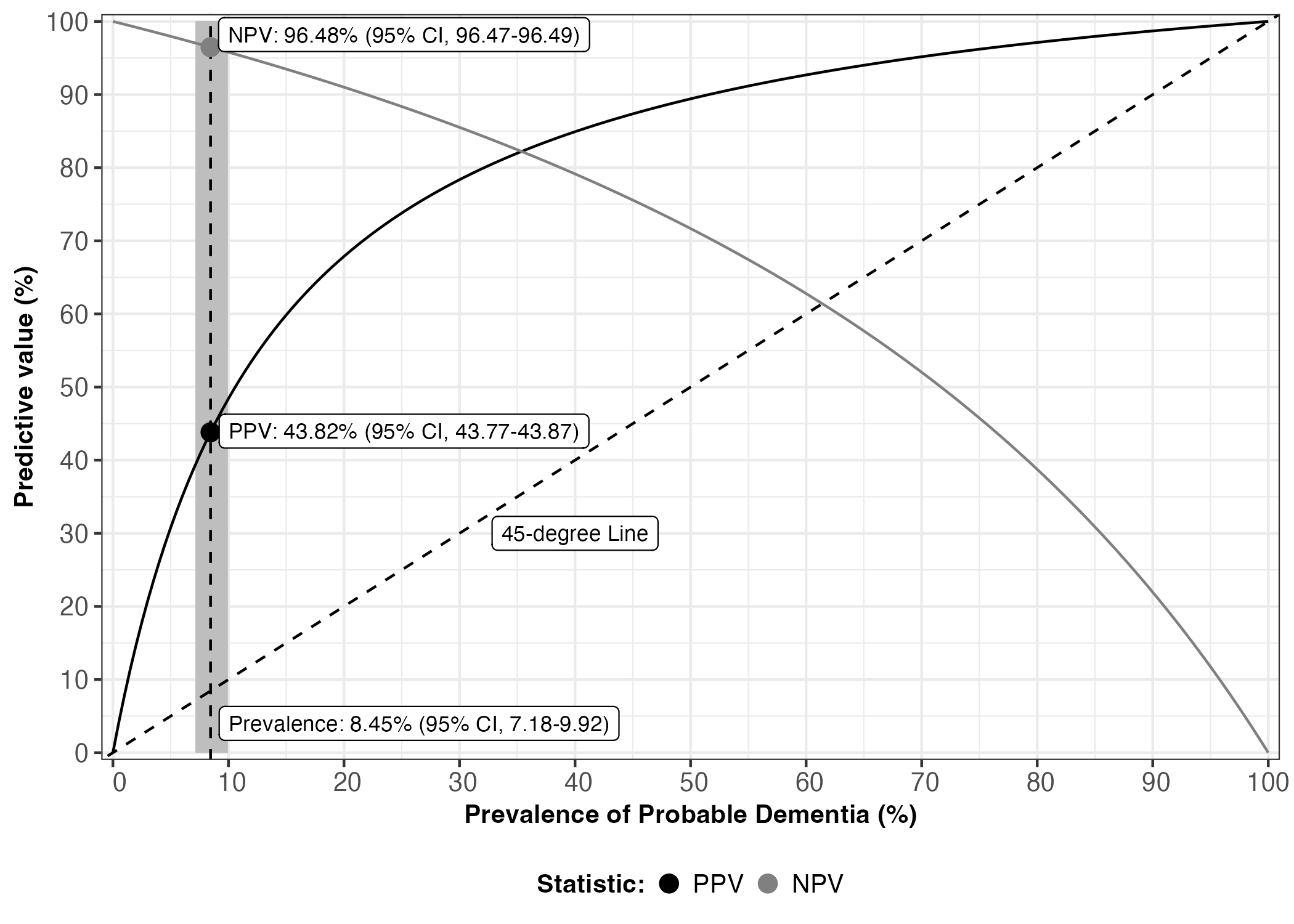 |
| **Abbreviations:** SCI, subjective cognitive impairment; NHATS, National Health and Aging Trends Study; PPV, positive predictive value; NPV, negative predictive value. |

- 1. **Odds ratio**

*The odds ratio (*$OR$*)* is another measure of the overall performance of a classifier. It is defined the usual way, as the ratio of the odds of the classifier producing a positive result among individuals with the condition (as assessed by the reference standard) to the odds of the classifier producing a positive result among individuals without the condition. Like the likelihood ratios, the $OR$ is also unaffected by the prevalence of the condition. The mathematical expression for the $OR$ is as follows:

| $OR = (a/b)/(c/d)= \frac{Sensitivity\times Specificity}{\left( 1-Specificity \right)\times\left( 1-Sensitivity \right)}$ | (16) |
| --- | --- |

The values of the $OR$ can range from zero to positive infinity. This statistic can be interpreted as the likelihood of a classifier yielding a positive result among individuals with the condition of interest, relative to those without the condition. As the value of the $OR$ becomes larger than 1, the discriminative power of the classifier also increases. An $OR$ larger than 1 is indicative of a higher likelihood of the classifier producing a positive result among individuals with the condition than among those without the condition. When the $OR$ is equal to unity, this means that the classifier does not contribute to discrimination, since the true positive rate is also equal to the false positive rate ($TPR = FPR$*)*. Finally, an $OR$ less than 1, indicates that the classifier is worse than chance at discriminating between cases with and without the condition.

There is a relationship between the $OR$ and the likelihood ratios. Indeed, the $OR$ is the ratio of the positive and negative likelihood ratios. To see this, it suffices to first notice that multiplying the prior odds of the condition ($Prevalence/[1 - Prevalence]$) by the $LR(+)$, yields the odds of the condition after a positive result, $O(+)$:

| $O\left( + \right)=\left( \frac{Prevalence}{1 - Prevalence} \right)\times LR\left( + \right)= \left( \frac{Prevalence}{1 - Prevalence} \right) \times\left( \frac{Sensitivity}{1 - Specificity} \right)$ | (17) |
| --- | --- |

Similarly, the odds of the condition following a negative result, $O(-)$, is the product of the prior odds of the condition and the $LR(-)$, as shown in the following equations:

| $O\left( - \right)=\left( \frac{Prevalence}{1 - Prevalence} \right)\times LR\left( - \right)= \left( \frac{Prevalence}{1 - Prevalence} \right) \times\left( \frac{1-Sensitivity}{Specificity} \right)$ | (18) |
| --- | --- |

Dividing the odds of the condition after a positive result, $O(+)$, by the odds after a negative result, $O(-)$, yields the $OR$:

| $OR=(a/b)/(c/d) =O(+)/O\left( - \right)= LR(+)/LR\left( - \right)$ | (19) |
| --- | --- |

- 1. **Receiver Operating Characteristic (ROC) Analysis and ROC curve (AUC)**

*The Receiver Operating Characteristic (*$ROC$*) Analysis* is a way to evaluate the performance of a classifier when the classification result is numerical, ordinal, or binary such as in our current application. Several types of $ROC$ analyses exist, including a two-way $ROC$ analysis when the reference standard has two categories (e.g., diseased vs healthy individuals), a three-way $ROC$ analysis when the reference standard has three categories (e.g., diseased, at-risk, and healthy individuals), and multi-class $ROC$ analysis when the reference standard involves more than three categories (e.g., mild, moderate, severe dementia). Two-way $ROC$ analysis is the most widely known and applied method, as is the case in our current application.

In most applications, classifiers produce real-valued scores corresponding with the strength of the prediction that a given case is positive. Translating these real-valued scores into binary predictions (e.g., Yes vs No) requires setting a cut-off, such that cases with scores above the selected cut-off are classified as positive, and cases with scores below the cut-off are classified as negative. Different cut-off values yield different levels of sensitivity and specificity. For example, a high cut-off will reduce the likelihood of classifying cases as positive, thus increasing the classifier’s specificity (i.e., $1 - FPR$) but reduce its sensitivity ($TPR$). Conversely, a low cut-off will increase the likelihood of classifying cases as positive, thus reducing the classifier’s specificity, but increasing its sensitivity. In our current application, there is no need for translating a score into a binary prediction from a predictive model constructed from training and validation samples, as all cases are already classified as positive or negative, based on responses to the Cognitive Disability item of the Census Bureau’s Disability Questionnaire. Thus, our analysis focuses only examining the empirical relation between the classifier (SCI) and the reference standard (validated NHATS probable dementia measure). We simply estimate the predicted probabilities of agreement between the SCI classification and the NHATS’s probable dementia classification. This approach allows us to trace the $ROC$ curve, further described below.

*The* $ROC$ *curve* is a graphical representation of the tradeoffs between sensitivity ($TPR$) and specificity ($1 - FPR$) of a binary classifier, across a range of cut-offs, to provide a wholistic picture of the spectrum of these tradeoffs, and to show the screening or diagnostic ability of the classifier.^12^ An ideal classifier would therefore have a very high sensitivity and a very high specificity, because it would be able to correctly identify true positive cases without misclassifying true negative cases. A perfectly random classifier is expected to produce points lying along the diagonal of the $ROC$ space, where the sensitivity is equal to the $FPR$. The closer the $ROC$ curve is to this 45-degree diagonal of the $ROC$ space, the less accurate the classifier. In contrast, classifiers that produce $ROC$ curves closer to the top-left corner are considered to have a better performance (in terms of sensitivity and specificity) than those farther away from that corner.

To evaluate the performance of a classifier against a reference standard, or compare alternative classifiers to each other, it can be useful to summarize the performance of each classifier into a single measure. One common approach is to calculate the area under the $ROC$ curve ($AUC$).

*The area under the* $ROC$ *curve (*$AUC$*)* provides an overall measure of the classifier’s performance in relation to the reference standard. For a binary classifier, the $AUC$ can be calculated as the average of the classifier’s sensitivity and specificity. The $AUC$ provides an idea about the benefit of using the classifier in question to rule in or out a condition of interest. The $AUC$ statistic can also be used to compare the performance of alternative classifiers. A classifier $A$ is considered superior to an alternative classifier $B$ if at all cut-offs, its $TPR$ exceeds and its $FPR$ is below those of classifier $B$. If this is the case, the $AUC$ for classifier $A$ will be larger than the $AUC$ for classifier $B$. In the case of a binary classifier, the $AUC$ measures the goodness of fit for the binary outcome in a logistic regression model. This is the precise definition of the $C$-statistic (also called “concordance” statistic or $C$-index). As a rule of thumb, the categorizations in eTable 5 can be used to describe an $ROC$ curve.

An extensive literature examines this topic, so readers interested in in-depth treatments of this topic should refer to this prior body of work.

| e**Figure 2: Receiver Operating Characteristic (ROC) curve of SCI against the NHATS probable dementia definition** |
| --- |
|  |
| **Abbreviations:** SCI, subjective cognitive impairment; NHATS, National Health and Aging Trends Study; ROC, receiver operator characteristic; AUC, area under the ROC curve; CI, confidence interval.  **Notes:** The ROC is constructed by estimating a probit of the SCI classification against the NHATS probable dementia classification, under the maximum likelihood procedure, and accounting for complex sampling design. We assume that the NHATS probable dementia definition to be the reference standard for dementia identification, as it is widely used in the literature to quantify dementia prevalence. We then assess the performance of the SCI measure against this NHATS probable dementia definition to assess the validity of SCI as a population-based dementia risk identification tool. Sensitivity is the probability that the classifier produces a positive result in individuals with the condition of interest, as defined in Eq. (8). Specificity is the probability that the classifier produces a negative result in individuals without the condition of interest, as defined in Eq. (9). The area under SCI curve (in solid black) is the area under the ROC curve (AUC) and measures the performance of SCI in correctly identifying individuals with dementia and those without dementia. ROC curves graphically relate true positive rates ($Sensitivity$) to false negative rates ($1- Specificity$) across a range of values. |

| **eTable 5: Benchmark scales for the area under the ROC curve (AUC)** | |
| --- | --- |
| **Area Under the ROC (AUC)** | **Interpretation^†^** |
| 0.9-1.0 | Very good |
| 0.8-0.9 | Good |
| 0.7-0.8 | Fair |
| 0.6-0.7 | Poor |
| 0.5-0.6 | Fail |
| **Abbreviations:** AUC, area under the ROC curve  **Notes:** ^†^ Hosme and Lemeshow (2000)^13^ and CLSI/NCCLS (2008)^14^ | |

1. **Additional results**

eTable 6 summarizes the estimated prevalence of dementia and SCI by subgroups. As anticipated, the inclusion of individuals meeting the possible dementia definition increased substantially the estimated prevalence of dementia. The remaining sections of this appendix report results from additional analyses.

Results from eTable 7 are qualitatively similar to those based on the restrictive measure of probable dementia only (Table 2), although levels of percent agreement and measures of agreement strength were lower. For example, agreement rates and the PABAK statistics were lower in older and less educated individuals (eTable 7).

eTable 8 summarizes the estimated associations between known risk factors for dementia and overall, false-positive, and false-negative misclassifications of probable dementia by SCI in Cox’s proportional hazard and modified Poisson models. These results are generally consistent with those from the main log-binomial model (Table 3). Misclassification rates are also higher in older and less educated individuals (eTable 9 and eTable 10). However, with the more liberal definition of dementia, misclassification rates varied by sex and race and ethnicity: rates were higher among males as well as non-Hispanic Blacks and Hispanics (eTable 9 and eTable 10).

The sensitivity and specificity of SCI against the NHATS possible or probable dementia definitions, by age group, sex, race and ethnicity, and education are summarized in eTable 11. The results suggest that SCI has low sensitivity, but high specificity against the NHATS possible or probable dementia definition. Sensitivity was highest in older and less educated individuals, females, and non-Hispanic Blacks. In contrast, specificity was highest in younger and more educated individuals, and non-Hispanics (eTable 11).

eTable 12 reports the PPV and NPV of SCI against the probable dementia and any dementia (possible or probable dementia) measures. The PPV for probable dementia was 43.8%, overall, and highest in the oldest age group (57.4%), females (48.6%), and in the lowest education group (48.5%); it was lowest in the youngest age group (26.7%), males (35.4%), Whites (40.3%), and the highest education category (26.4%). NPV was 96.5% overall, and highest for the youngest age group (98.4%), females (96.5%), Whites (97.8%), and those with some college education (98.9%), and lowest in the oldest age group (91.2%), Other race and ethnicity (89.1%), and in the lowest education category (91.5%).

eTable 13 reports additional measures of the accuracy of SCI against the NHATS dementia definitions, by age group, sex, race and ethnicity, and education. The odds of SCI among individuals with probable dementia, relative to those without probable dementia was 21.4, with an AUC of 0.78, indicating a fair to good discriminatory performance of SCI for dementia (eTable 12). ORs and AUCs were highest in the youngest age group (OR, 22.9; AUC, 0.75), females (OR, 26.0; AUC, 0.80), Whites (OR, 23.6; AUC, 0.79), and those high school graduates (OR, 30.8; AUC, 0.81) (eTable 12).

| **eTable 6: Prevalence of dementia and SCI among NHATS participants in 2012** | | | | | | | | | | | | | |
| --- | --- | --- | --- | --- | --- | --- | --- | --- | --- | --- | --- | --- | --- |
|  | **Probable Dementia**  **(Weighted N=2,999,053)** | | |  | **Possible or Probable Dementia**  **(Weighted N=8,909,264)** | | |  | **SCI**  **(Weighted N = 4,342,883)** | | | |  |
|  | **Total** | **SCI** | **No SCI** |  | **Total** | **SCI** | **No SCI** |  | **Total** | **Probable Dementia** | **Possible/Probable**  **Dementia** | **No**  **Dementia** |  |
| **Characteristics** | **% (SE)** | **% (SE)** | **% (SE)** |  | **% (SE)** | **% (SE)** | **% (SE)** |  | **% (SE)** | **% (SE)** | **% (SE)** | **% (SE)** |  |
| **Overall*** | 8.45 (0.68) | 43.82 (3.59) | 3.52 (0.53) |  | 25.10 (1.49) | 68.55 (3.60) | 19.05 (1.45) |  | 12.24 (0.85) | 63.45 (4.03) | 33.42 (2.37) | 5.14 (0.72) |  |
| **Age group (y)** |  |  |  |  |  |  |  |  |  |  |  |  |  |
| 65-74 | 3.26 (0.65) | 26.68 (6.83) | 1.56 (0.52) |  | 13.82 (1.61) | 44.60 (7.48) | 11.59 (1.55) |  | 6.75 (0.87) | 55.29 (12.09) | 21.80 (4.29) | 4.34 (0.78) |  |
| 75-84 | 10.55 (1.24) | 45.36 (4.92) | 4.38 (0.93) |  | 29.34 (2.19) | 73.43 (4.69) | 21.54 (2.19) |  | 15.04 (1.69) | 64.70 (5.78) | 37.64 (3.81) | 5.66 (1.29) |  |
| ≥ 85 | 20.08 (2.30) | 57.37 (6.23) | 8.85 (1.72) |  | 51.11 (2.79) | 83.39 (4.21) | 41.38 (3.00) |  | 23.16 (2.11) | 66.15 (5.06) | 37.78 (3.27) | 7.87 (2.07) |  |
| **Sex** |  |  |  |  |  |  |  |  |  |  |  |  |  |
| Male | 6.78 (0.82) | 35.38 (4.84) | 3.53 (0.75) |  | 25.32 (1.74) | 62.46 (5.29) | 21.09 (1.88) |  | 10.22 (1.04) | 53.34 (6.52) | 25.22 (2.88) | 5.14 (1.02) |  |
| Female | 9.72 (0.99) | 48.59 (4.40) | 3.51 (0.61) |  | 24.94 (1.92) | 72.00 (4.04) | 17.42 (1.75) |  | 13.77 (1.23) | 68.84 (4.04) | 39.76 (3.19) | 5.14 (0.88) |  |
| **Race and ethnicity** |  |  |  |  |  |  |  |  |  |  |  |  |  |
| White, non-Hispanic | 7.00 (0.66) | 40.27 (4.36) | 2.77 (0.52) |  | 21.20 (1.64) | 64.90 (4.42) | 15.65 (1.54) |  | 11.27 (0.90) | 64.85 (5.01) | 34.52 (2.93) | 5.02 (0.76) |  |
| Black, non-Hispanic | 12.73 (2.13) | 47.33 (7.03) | 5.50 (1.33) |  | 37.08 (2.44) | 81.82 (3.99) | 27.73 (2.85) |  | 17.27 (2.26) | 64.24 (8.25) | 38.12 (5.16) | 4.99 (1.24) |  |
| Hispanic | 15.73 (3.23) | 54.70 (8.92) | 6.17 (1.96) |  | 47.22 (5.85) | 73.37 (8.34) | 40.80 (6.77) |  | 19.70 (3.08) | 68.52 (8.12) | 30.62 (6.12) | 9.94 (3.24) |  |
| Other, non-Hispanic | 17.51 (5.56) | 80.01 (11.63) | 10.95 (4.72) |  | 44.51 (8.18) | 91.92 (8.11) | 39.52 (8.15) |  | 9.51 (3.39) | 43.44 (13.05) | 19.64 (6.49) | 1.39 (1.40) |  |
| **Education** |  |  |  |  |  |  |  |  |  |  |  |  |  |
| Less than high school | 17.88 (1.88) | 48.51 (4.98) | 8.50 (1.46) |  | 48.04 (2.76) | 74.28 (4.79) | 40.00 (3.11) |  | 23.45 (2.33) | 63.62 (5.08) | 36.26 (3.41) | 11.60 (2.62) |  |
| High school graduate | 8.25 (0.90) | 47.91 (5.98) | 2.90 (0.62) |  | 24.29 (1.91) | 72.91 (5.58) | 17.73 (1.80) |  | 11.89 (1.36) | 69.07 (5.58) | 35.69 (3.61) | 4.26 (1.09) |  |
| Some college | 5.54 (1.36) | 36.00 (11.36) | 2.26 (0.87) |  | 18.52 (2.06) | 56.03 (11.70) | 14.48 (2.22) |  | 9.72 (1.59) | 63.22 (12.52) | 29.41 (7.07) | 5.25 (1.68) |  |
| College graduate and higher | 3.39 (0.76) | 26.42 (8.24) | 2.00 (0.67) |  | 12.87 (1.53) | 52.65 (10.50) | 10.47 (1.51) |  | 5.69 (1.03) | 44.40 (13.15) | 23.28 (5.50) | 3.09 (0.93) |  |
| **Abbreviations:** SCI, subjective cognitive impairment; NHATS, National Health and Aging Trends Study.  **Notes:**  *Total sample included n=1,936 observations (weighted n=35,489,497). All estimates are adjusted using Round 2 analytic weights, to produce a nationally representative sample and account for differential probabilities of selection and nonresponse in NHATS and were further adjusted to reflect the fact that the analytic sample is a random one-third sample of NHATS participants in 2012. | | | | | | | | | | | | | |

| **eTable 7: Percent agreement and strength of agreement between SCI and any dementia (NHATS possible or probable dementia definitions), overall, and by age group, sex, race and ethnicity, and education** | | | | | | | | |  |
| --- | --- | --- | --- | --- | --- | --- | --- | --- | --- |
|  | **Percent Agreement, %** | |  | **Indices, %** | |  | **Agreement Strength, Est. (95% CI)** | | |
| **Characteristics** | **Observed (**$\boldsymbol{p}_{\boldsymbol{o}}$**)^¶^** | **Expected (**$\boldsymbol{p}_{\boldsymbol{e}}$**) ^¶^** |  | **Prevalence (**$\boldsymbol{PI}$**) ^†^** | **Bias (**$\boldsymbol{BI}$**) ^†^** |  | **Kappa (**$\boldsymbol{\kappa}$**)^‡^** | **PABAK (**$\boldsymbol{\kappa}^{\boldsymbol{*}}$**) ^‡^** | |
| **Overall** | 79.40 | 68.80 |  | -62.70 | -12.90 |  | 0.34 (0.29-0.39) | 0.59 (0.55-0.62) | |
| **Age group (y)** |  |  |  |  |  |  |  |  | |
| 65-74 | 85.45 | 81.29 |  | -79.42 | -7.07 |  | 0.22 (0.12-0.32) | 0.71 (0.66-0.76) | |
| 75-84 | 77.71 | 64.44 |  | -55.62 | -14.30 |  | 0.37 (0.30-0.44) | 0.55 (0.50-0.61) | |
| ≥85 | 64.35 | 49.40 |  | -25.73 | -27.95 |  | 0.30 (0.22-0.37) | 0.29 (0.20-0.37) | |
| **Sex** |  |  |  |  |  |  |  |  | |
| Male | 77.23 | 69.63 |  | -64.46 | -15.10 |  | 0.25 (0.18-0.32) | 0.54 (0.49-0.60) | |
| Female | 81.12 | 68.16 |  | -61.29 | -11.17 |  | 0.41 (0.34-0.47) | 0.62 (0.58-0.67) | |
| **Race and ethnicity** |  |  |  |  |  |  |  |  | |
| White, non-Hispanic | 82.16 | 72.31 |  | -67.53 | -9.93 |  | 0.36 (0.29-0.42) | 0.64 (0.60-0.68) | |
| Black, non-Hispanic | 73.91 | 58.46 |  | -45.65 | -19.80 |  | 0.37 (0.28-0.46) | 0.48 (0.39-0.56) | |
| Hispanic | 61.99 | 51.69 |  | -33.08 | -27.51 |  | 0.21 (0.06-0.37) | 0.24 (0.06-0.42) | |
| **Education** |  |  |  |  |  |  |  |  | |
| Less than high school | 63.35 | 51.04 |  | -28.51 | -24.59 |  | 0.25 (0.18-0.33) | 0.27 (0.18-0.35) | |
| High school graduate | 81.16 | 69.59 |  | -63.81 | -12.40 |  | 0.38 (0.30-0.46) | 0.62 (0.56-0.68) | |
| Some college | 82.65 | 75.36 |  | -71.76 | -8.80 |  | 0.30 (0.16-0.43) | 0.65 (0.57-0.73) | |
| College graduate and higher | 87.43 | 82.90 |  | -81.44 | -7.18 |  | 0.26 (0.13-0.40) | 0.75 (0.69-0.81) | |
| **Abbreviations:** NHATS, National Health and Aging Trends Study; SCI, subjective cognitive impairment; CI, confidence interval; y, years; PABAK, prevalence-and bias-adjusted kappa.  **Notes:** Sample included n=1,936 observations (weighted n=35,489,497). Other race and ethnicity category is not reported due to the small sample size (unweighted n=73). All estimates are adjusted using Round 2 analytic weights, to produce a nationally representative sample and account for differential probabilities of selection and nonresponse in NHATS and were further adjusted to reflect the fact that the analytic sample is a random one-third sample of NHATS participants in 2012.  ^¶^ Percent observed agreement ($p_{o}$) is defined as how much agreement is actually present or “observed” and is calculated as $\mathrm{QUOTE}p_{0}=(a+d)/N p_{o}=(a+d)/N$ where$a$ $a$ and $d$ represent the frequencies in which the two instruments agree ($a$ $a$ $a$ is when both instruments say “Yes” and denotes the true-positive cases, TP; $d$ when both instruments say “No” and denotes the true-negative cases, TN) and $QUOTE N N$ is the total frequency of observation (eTable 3). Percent expected agreement ($p_{e}$)$p_{e}$ is the agreement that is expected to be present by chance alone and is calculated as $p_{e}=\left( \left( \left( a+b \right)\times\left( a+c \right) \right)+\left( \left( c+d \right)\times\left( b+d \right) \right) \right)/N^{2}$ where$a$ $a$ and $d$ are defined as before in note ^¶^;$a$ $b$ and $c$ represent represent the frequencies in which the two instruments do not agree ($a$ $a$ $b$ denotes the false-positive cases, FP; $c$ denotes the false-negative cases, FN) and $N$ $QUOTE N N$ is the total frequency of observation.  ^†^ The prevalence index ($PI$) is the difference between the prevalence of TP and TN ratings. It is calculated as $PI=\left( a-d \right)/N$. A positive value of the index indicates that the prevalence of TP ratings exceeds that of TN ratings; a negative signage indicates the opposite relationship. The bias index ($BI$) represents the difference between the prevalence of FP and FN ratings. It is calculated as $BI=(b-c)/N$. A positive sign indicates that the prevalence of FP ratings exceeds that of FN ratings, with a negative signage indicating the opposite relationship.  ^‡^ Kappa ($\kappa$) denotes the Cohen and Conger’s kappa and is calculated as $\kappa^{*}=2p_{0}-1$ $\kappa=(p_{o}-p_{e})/(1-p_{e})$. PABAK ($\kappa^{*}$) represents the Brennan and Prediger coefficient and is calculated as $\kappa^{*}=2p_{0}-1$ $\kappa^{*}=2p_{o}-1$. The extent of agreement is interpreted from benchmark scales in Landis and Kock (1977) represented in eTable 4. | | | | | | | | |  |

| **eTable 8: Predictors of overall, false-positive, and false-negative misclassifications of probable dementia by SCI in Cox’s proportional hazard and modified Poisson models** | | | | | | | | | | | | | | | | | |
| --- | --- | --- | --- | --- | --- | --- | --- | --- | --- | --- | --- | --- | --- | --- | --- | --- | --- |
|  | **Cox’s Proportional Hazard Models** | | | | | | | |  | **Modified Poisson Models** | | | | | | | |
|  | **Overall Misclassification** | |  | **False-Positive Misclassification** | |  | **False-Negative Misclassification** | |  | **Overall Misclassification** | |  | **False-Positive Misclassification** | |  | **False-Negative Misclassification** | |
| **Predictors of Misclassification** | **HR** | **SE** |  | **HR** | **SE** |  | **HR** | **SE** |  | **RR** | **SE** |  | **RR** | **SE** |  | **RR** | **SE** |
| **Intercept** | -- | -- |  | -- | -- |  | -- | -- |  | 0.119*** | (0.026) |  | 0.104*** | (0.027) |  | 0.502* | (0.185) |
| **Age group, y (ref = 65-74 y)** |  |  |  |  |  |  |  |  |  |  |  |  |  |  |  |  |  |
| 75-84 | 1.753*** | (0.269) |  | 1.673*** | (0.322) |  | 0.855 | (0.278) |  | 1.753*** | (0.269) |  | 1.673*** | (0.322) |  | 0.855 | (0.278) |
| ≥85 | 2.423*** | (0.478) |  | 2.228*** | (0.566) |  | 0.825 | (0.259) |  | 2.423*** | (0.478) |  | 2.228*** | (0.566) |  | 0.825 | (0.259) |
| **Female (ref = Male)** | 0.905 | (0.140) |  | 0.982 | (0.179) |  | 0.683* | (0.141) |  | 0.905 | (0.140) |  | 0.982 | (0.179) |  | 0.683* | (0.141) |
| **Race and ethnicity (ref = White, non-Hispanic)** | | | | | | | | | | | | | | | | | |
| Black, non-Hispanic | 1.220 | (0.202) |  | 1.161 | (0.231) |  | 0.958 | (0.270) |  | 1.220 | (0.202) |  | 1.161 | (0.231) |  | 0.958 | (0.270) |
| Hispanic | 1.086 | (0.261) |  | 1.005 | (0.301) |  | 0.887 | (0.338) |  | 1.086 | (0.261) |  | 1.005 | (0.301) |  | 0.887 | (0.338) |
| **Education (ref = Less than high school)** | | | | | | | | | | | | | | | | | |
| High school graduate | 0.523*** | (0.105) |  | 0.472*** | (0.110) |  | 0.953 | (0.254) |  | 0.523*** | (0.105) |  | 0.472*** | (0.110) |  | 0.953 | (0.254) |
| Some college | 0.475*** | (0.131) |  | 0.450** | (0.149) |  | 1.016 | (0.410) |  | 0.475*** | (0.131) |  | 0.450** | (0.149) |  | 1.016 | (0.410) |
| College graduate and higher | 0.383*** | (0.090) |  | 0.325*** | (0.098) |  | 1.324 | (0.451) |  | 0.383*** | (0.090) |  | 0.325*** | (0.098) |  | 1.324 | (0.451) |
| **Observations** |  |  |  |  |  |  |  |  |  |  |  |  |  |  |  |  |  |
| Unweighted | 1,863 | |  | 1,660 | |  | 203 | |  | 1,863 | |  | 1,660 | |  | 203 | |
| Weighted | 34,076,757 | |  | 31,325,138 | |  | 2,751,618 | |  | 34,076,757 | |  | 31,325,138 | |  | 2,751,618 | |
| **Number of strata** | 56 | |  | 56 | |  | 52 | |  | 56 | |  | 56 | |  | 52 | |
| **Number of PSUs** | 112 | |  | 112 | |  | 104 | |  | 112 | |  | 112 | |  | 104 | |
| **Degrees of freedom** | 49 | |  | 49 | |  | 45 | |  | 49 | |  | 49 | |  | 45 | |
| **Adjusted Wald test F-statistic** | 9.26*** | |  | 6.40*** | |  | 1.10 | |  | -- | |  | -- | |  | -- | |
| **Abbreviations:** NHATS, National Health and Aging Trends Study; SCI, subjective cognitive impairment; HR, hazard ratio; RR, relative risk; SE, standard error.  **Notes:** Estimates are from Cox’s proportional hazard and modified Poisson models. Sample included n=1,863 observations (weighted n=34,076,757). Other race and ethnicity category is not reported due to the small sample size (unweighted n=73). All estimates are adjusted using Round 2 analytic weights, to produce a nationally representative sample and account for differential probabilities of selection and nonresponse in NHATS and were further adjusted to reflect the fact that the analytic sample is a random one-third sample of NHATS participants in 2012. SE in parentheses; statistical significance: *** p<0.01, ** p<0.05, * p<0.1. | | | | | | | | | | | | | | | | | |

| **eTable 9: Predictors of overall, false-positive, and false-negative misclassifications of any dementia (possible or probable dementia) by SCI in logistic regression and log-binomial models** | | | | | | | | | | | | | | | | | | |
| --- | --- | --- | --- | --- | --- | --- | --- | --- | --- | --- | --- | --- | --- | --- | --- | --- | --- | --- |
|  | **Logistic Regression Models** | | | | | | | |  | **Log-binomial Models** | | | | | | | | |
|  | **Overall Misclassification** | |  | **False-Positive Misclassification** | |  | **False-Negative Misclassification** | |  | **Overall Misclassification** | |  | **False-Positive Misclassification** | |  | **False-Negative Misclassification** | | |
| **Predictors of Misclassification** | **OR** | **SE** |  | **OR** | **SE** |  | **OR** | **SE** |  | **RR** | **SE** |  | **RR** | **SE** |  | **RR** | **SE** |  |
| **Intercept** | 0.363*** | (0.070) |  | 0.120*** | (0.042) |  | 4.120*** | (1.576) |  | 0.246*** | (0.035) |  | 0.107*** | (0.035) |  | 0.782** | (0.093) |  |
| **Age group, y (ref = 65-74 y)** |  |  |  |  |  |  |  |  |  |  |  |  |  |  |  |  |  |  |
| 75-84 | 1.608*** | (0.231) |  | 1.257 | (0.354) |  | 0.457** | (0.146) |  | 1.409*** | (0.160) |  | 1.217 | (0.326) |  | 0.769*** | (0.070) |  |
| ≥85 | 3.393*** | (0.556) |  | 1.657 | (0.637) |  | 0.478** | (0.146) |  | 2.203*** | (0.277) |  | 1.522 | (0.542) |  | 0.812** | (0.071) |  |
| **Female (ref = Male)** | 0.647*** | (0.089) |  | 0.936 | (0.259) |  | 0.563** | (0.123) |  | 0.755*** | (0.068) |  | 0.948 | (0.242) |  | 0.819** | (0.064) |  |
| **Race and ethnicity (ref = White, non-Hispanic)** | | | | | | | | | | | | | | | | | | |
| Black, non-Hispanic | 1.398** | (0.219) |  | 0.798 | (0.266) |  | 0.832 | (0.231) |  | 1.221** | (0.120) |  | 0.803 | (0.247) |  | 0.956 | (0.103) |  |
| Hispanic | 2.114*** | (0.557) |  | 1.334 | (0.596) |  | 1.160 | (0.422) |  | 1.542*** | (0.199) |  | 1.289 | (0.504) |  | 1.078 | (0.143) |  |
| **Education (ref = Less than high school)** | | | | | | | | | | | | | | | | | | |
| High school graduate | 0.509*** | (0.088) |  | 0.341*** | (0.136) |  | 1.131 | (0.215) |  | 0.661*** | (0.077) |  | 0.373*** | (0.138) |  | 1.138 | (0.114) |  |
| Some college | 0.466*** | (0.101) |  | 0.408* | (0.198) |  | 1.503 | (0.619) |  | 0.604*** | (0.095) |  | 0.441* | (0.197) |  | 1.234 | (0.177) |  |
| College graduate and higher | 0.296*** | (0.064) |  | 0.252*** | (0.112) |  | 1.559 | (0.592) |  | 0.425*** | (0.070) |  | 0.280*** | (0.119) |  | 1.153 | (0.150) |  |
| **Observations** |  |  |  |  |  |  |  |  |  |  |  |  |  |  |  |  |  |  |
| Unweighted | 1,863 | |  | 1,284 | |  | 579 | |  | 1,863 | |  | 1,284 | |  | 579 | | |
| Weighted | 34,076,757 | |  | 25,796,243 | |  | 8,280,514 | |  | 34,076,757 | |  | 25,796,243 | |  | 8,280,514 | | |
| **Number of strata** | 56 | |  | 56 | |  | 52 | |  | 56 | |  | 56 | |  | 52 | | |
| **Number of PSUs** | 112 | |  | 112 | |  | 104 | |  | 112 | |  | 112 | |  | 104 | | |
| **Degrees of freedom** | 49 | |  | 49 | |  | 49 | |  | 49 | |  | 49 | |  | 49 | | |
| **Adjusted Wald test F-statistic** | 12.04*** | |  | 2.95*** | |  | 2.61** | |  | -- | |  | -- | |  | -- | | |
| **Abbreviations:** NHATS, National Health and Aging Trends Study; SCI, subjective cognitive impairment; OR, odds ratio; RR, relative risk; SE, standard error.  **Notes:** Estimates are from logistic regression and log-binomial models. Sample included n=1,863 observations (weighted n=34,076,757). Other race and ethnicity category is not reported due to the small sample size (unweighted n=73). All estimates are adjusted using Round 2 analytic weights, to produce a nationally representative sample and account for differential probabilities of selection and nonresponse in NHATS and were further adjusted to reflect the fact that the analytic sample is a random one-third sample of NHATS participants in 2012. SE in parentheses; statistical significance: *** p<0.01, ** p<0.05, * p<0.1. | | | | | | | | | | | | | | | | | | |

| **eTable 10: Predictors of overall, false-positive, and false-negative misclassifications of any dementia (possible or probable dementia) by SCI in Cox’s proportional hazard and modified Poisson models** | | | | | | | | | | | | | | | | | |
| --- | --- | --- | --- | --- | --- | --- | --- | --- | --- | --- | --- | --- | --- | --- | --- | --- | --- |
|  | **Cox’s Proportional Hazard Models** | | | | | | | |  | **Modified Poisson Models** | | | | | | | |
|  | **Overall Misclassification** | |  | **False-Positive Misclassification** | |  | **False-Negative Misclassification** | |  | **Overall Misclassification** | |  | **False-Positive Misclassification** | |  | **False-Negative Misclassification** | |
| **Predictors of Misclassification** | **OR** | **SE** |  | **OR** | **SE** |  | **OR** | **SE** |  | **HR** | **SE** |  | **HR** | **SE** |  | **HR** | **SE** |
| **Intercept** | -- | -- |  | -- | -- |  | -- | -- |  | 0.251*** | (0.036) |  | 0.106*** | (0.034) |  | 0.804** | (0.0818) |
| **Age group, y (ref = 65-74 y)** |  |  |  |  |  |  |  |  |  |  |  |  |  |  |  |  |  |
| 75-84 | 1.452*** | (0.166) |  | 1.238 | (0.326) |  | 0.796** | (0.071) |  | 1.452*** | (0.166) |  | 1.238 | (0.326) |  | 0.796** | (0.071) |
| ≥85 | 2.437*** | (0.295) |  | 1.593 | (0.563) |  | 0.810** | (0.067) |  | 2.437*** | (0.295) |  | 1.593 | (0.563) |  | 0.810** | (0.067) |
| **Female (ref = Male)** | 0.724*** | (0.072) |  | 0.939 | (0.242) |  | 0.831** | (0.060) |  | 0.724*** | (0.072) |  | 0.939 | (0.242) |  | 0.831** | (0.060) |
| **Race and ethnicity (ref = White, non-Hispanic)** | | | | | | | | | | | | | | | | | |
| Black, non-Hispanic | 1.270** | (0.137) |  | 0.813 | (0.251) |  | 0.942 | (0.090) |  | 1.270** | (0.137) |  | 0.813 | (0.251) |  | 0.942 | (0.090) |
| Hispanic | 1.653*** | (0.261) |  | 1.294 | (0.516) |  | 1.059 | (0.120) |  | 1.653*** | (0.261) |  | 1.294 | (0.516) |  | 1.059 | (0.120) |
| **Education (ref = Less than high school)** | | | | | | | | | | | | | | | | | |
| High school graduate | 0.623*** | (0.075) |  | 0.371*** | (0.136) |  | 1.043 | (0.070) |  | 0.623*** | (0.075) |  | 0.371*** | (0.136) |  | 1.043 | (0.0702) |
| Some college | 0.581*** | (0.094) |  | 0.441* | (0.197) |  | 1.141 | (0.147) |  | 0.581*** | (0.094) |  | 0.441* | (0.197) |  | 1.141 | (0.147) |
| College graduate and higher | 0.399*** | (0.067) |  | 0.277*** | (0.115) |  | 1.142 | (0.125) |  | 0.399*** | (0.067) |  | 0.277*** | (0.115) |  | 1.142 | (0.125) |
| **Observations** |  |  |  |  |  |  |  |  |  |  |  |  |  |  |  |  |  |
| Unweighted | 1,863 | |  | 1,284 | |  | 579 | |  | 1,863 | |  | 1,284 | |  | 579 | |
| Weighted | 34,076,757 | |  | 25,796,243 | |  | 8,280,514 | |  | 34,076,757 | |  | 25,796,243 | |  | 8,280,514 | |
| **Number of strata** | 56 | |  | 56 | |  | 52 | |  | 56 | |  | 56 | |  | 52 | |
| **Number of PSUs** | 112 | |  | 112 | |  | 104 | |  | 112 | |  | 112 | |  | 104 | |
| **Degrees of freedom** | 49 | |  | 49 | |  | 49 | |  | 49 | |  | 49 | |  | 49 | |
| **Adjusted Wald test F-statistic** | 13.89*** | |  | 3.13*** | |  | 3.27*** | |  | -- | |  | -- | |  | -- | |
| **Abbreviations:** NHATS, National Health and Aging Trends Study; SCI, subjective cognitive impairment; OR, odds ratio; HR, hazard ratio; SE, standard error.  **Notes:** Estimates are from logistic regression and Cox’s proportional hazard models. Sample included n=1,863 observations (weighted n=34,076,757). Other race and ethnicity category is not reported due to the small sample size (unweighted n=73). All estimates are adjusted using Round 2 analytic weights, to produce a nationally representative sample and account for differential probabilities of selection and nonresponse in NHATS and were further adjusted to reflect the fact that the analytic sample is a random one-third sample of NHATS participants in 2012. SE in parentheses; statistical significance: *** p<0.01, ** p<0.05, * p<0.1. | | | | | | | | | | | | | | | | | |

| **eTable 11: Sensitivity and specificity of SCI against the NHATS possible or probable dementia definitions, by age group, sex, race and ethnicity, and education** | | |
| --- | --- | --- |
| **Characteristic** | **Sensitivity** ^†^  **(95% CI)** | **Specificity** ^‡^  **(95% CI)** |
| **Overall** | 33.42 (33.39 - 33.45) | 94.86 (94.85 - 94.87) |
| **Age group (y)** |  |  |
| 65-74 | 21.80 (21.74 - 21.85) | 95.66 (95.65 - 95.67) |
| 75-84 | 37.64 (37.59 - 37.69) | 94.34 (94.33 - 94.36) |
| ≥85 | 37.78 (37.73 - 37.84) | 92.13 (92.10 - 92.17) |
| **Sex** |  |  |
| Male | 25.22 (25.17 - 25.26) | 94.86 (94.85 - 94.87) |
| Female | 39.76 (39.72 - 39.81) | 94.86 (94.85 - 94.87) |
| **Race and ethnicity** |  |  |
| White, non-Hispanic | 34.52 (34.48 - 34.55) | 94.98 (94.97 - 94.99) |
| Black, non-Hispanic | 38.12 (38.02 - 38.21) | 95.01 (94.98 - 95.04) |
| Hispanic | 30.62 (30.53 - 30.71) | 90.06 (90.01 - 90.11) |
| Other, non-Hispanic | 19.64 (19.54 - 19.74) | 98.61 (98.59 - 98.64) |
| **Education** |  |  |
| Less than high school | 36.26 (36.21 - 36.31) | 88.40 (88.36 - 88.43) |
| High school graduate | 35.69 (35.64 - 35.75) | 95.74 (95.73 - 95.76) |
| Some college | 29.41 (29.33 - 29.49) | 94.75 (94.73 - 94.77) |
| College graduate and higher | 23.28 (23.21 - 23.36) | 96.91 (96.90 - 96.92) |
| **Abbreviations:** NHATS, National Health and Aging Trends Study; SCI, subjective cognitive impairment; CI, confidence interval; y, years  **Notes:** Sample includes n=1,936 observations (weighted n=35,489,497). All estimates are adjusted using Round 2 analytic weights, to produce a nationally representative sample and account for differential probabilities of selection and nonresponse in NHATS and were further adjusted to reflect the fact that the analytic sample is a random one-third sample of NHATS participants in 2012. We assume that the NHATS probable dementia definition to be the reference standard for dementia identification, as it is widely used in the literature to quantify dementia prevalence. We then assess the performance of the SCI measure against this NHATS probable dementia definition to assess the validity of SCI as a population-based dementia risk identification tool.  ^†^ Sensitivity is the probability that the classifier produces a positive result in individuals with the condition of interest.  ^‡^ Specificity is the probability that the classifier produces a negative result in individuals without the condition of interest. | | |

| **eTable 12: Predictive values of SCI against the NHATS dementia definitions, by age group, sex, race and ethnicity, and education** | | | | | |
| --- | --- | --- | --- | --- | --- |
|  | **Probable Dementia** | |  | **Possible or Probable Dementia** | |
| **Characteristic** | **PPV ^†^**  **(95% CI)** | **NPV** ^§^  **(95% CI)** |  | **PPV ^†^**  **(95% CI)** | **NPV** ^§^  **(95% CI)** |
| **Overall** | 43.82 (43.77 - 43.87) | 96.48 (96.47 - 96.49) |  | 68.55 (68.51 - 68.60) | 80.95 (80.94 - 80.97) |
| **Age group (y)** |  |  |  |  |  |
| 65-74 | 26.68 (26.60 - 26.76) | 98.44 (98.43 - 98.44) |  | 44.60 (44.51 - 44.69) | 88.41 (88.39 - 88.42) |
| 75-84 | 45.36 (45.29 - 45.44) | 95.62 (95.61 - 95.63) |  | 73.43 (73.37 - 73.50) | 78.46 (78.44 - 78.49) |
| ≥85 | 57.37 (57.28 - 57.45) | 91.15 (91.12 - 91.18) |  | 83.39 (83.33 - 83.46) | 58.62 (58.57 - 58.66) |
| **Sex** |  |  |  |  |  |
| Male | 35.38 (35.31 - 35.46) | 96.47 (96.47 - 96.48) |  | 62.46 (62.39 - 62.54) | 78.91 (78.89 - 78.93) |
| Female | 48.59 (48.54 - 48.65) | 96.49 (96.48 - 96.49) |  | 72.00 (71.95 - 72.05) | 82.58 (82.56 - 82.60) |
| **Race and ethnicity** |  |  |  |  |  |
| White, non-Hispanic | 40.27 (40.22 - 40.32) | 97.23 (97.22 - 97.23) |  | 64.90 (64.85 - 64.96) | 84.35 (84.34 - 84.37) |
| Black, non-Hispanic | 47.33 (47.20 - 47.47) | 94.50 (94.47 - 94.53) |  | 81.82 (81.71 - 81.92) | 72.27 (72.21 - 72.32) |
| Hispanic | 54.70 (54.56 - 54.85) | 93.83 (93.80 - 93.87) |  | 73.37 (73.24 - 73.50) | 59.20 (59.13 - 59.27) |
| Other, non-Hispanic | 80.01 (79.79 - 80.22) | 89.05 (89.00 - 89.11) |  | 91.92 (91.77 - 92.06) | 60.48 (60.39 - 60.56) |
| **Education** |  |  |  |  |  |
| Less than high school | 48.51 (48.43 - 48.58) | 91.50 (91.48 - 91.53) |  | 74.28 (74.22 - 74.35) | 60.00 (59.96 - 60.04) |
| High school graduate | 47.91 (47.83 - 47.99) | 97.10 (97.09 - 97.11) |  | 72.91 (72.84 - 72.98) | 82.27 (82.25 - 82.29) |
| Some college | 36.00 (35.88 - 36.12) | 97.74 (97.73 - 97.76) |  | 56.03 (55.90 - 56.15) | 85.52 (85.49 - 85.55) |
| College graduate and higher | 26.42 (26.30 - 26.54) | 98.00 (97.99 - 98.01) |  | 52.65 (52.51 - 52.79) | 89.53 (89.51 - 89.55) |
| **Abbreviations:** SCI, subjective cognitive impairment; NHATS, National Health and Aging Trends Study; CI, confidence interval; y, years; PPV, positive predictive value; NPV, negative predictive value.  **Notes:** Sample includes n=1,936 observations (weighted n=35,489,497). All estimates are adjusted using Round 2 analytic weights, to produce a nationally representative sample and account for differential probabilities of selection and nonresponse in NHATS and were further adjusted to reflect the fact that the analytic sample is a random one-third sample of NHATS participants in 2012. We assume that the NHATS probable dementia definition to be the reference standard for dementia identification, as it is widely used in the literature to quantify dementia prevalence. We then assess the performance of the SCI measure against this NHATS probable dementia definition to assess the validity of SCI as a population-based dementia risk identification tool.  **^†^** Positive predictive value (PPV) is a measure of precision and represents the probability that a patient with a positive test truly has the condition.  ^§^ Negative predictive value (NPV) is the probability that a patient with a negative test truly does not have the condition. | | | | | |

| **eTable 13: Additional measures of the accuracy of SCI against the NHATS dementia definitions, by age group, sex, race and ethnicity, and education** | | | | | | | | | |
| --- | --- | --- | --- | --- | --- | --- | --- | --- | --- |
|  | **Probable Dementia** | | | |  | **Possible or Probable Dementia** | | | |
| **Characteristic** | **LR(+)**  **(95% CI)** | **LR(-)**  **(95% CI)** | **OR^*^**  **(95% CI)** | **AUC** ^¶^  **(95% CI)** |  | **LR(+)**  **(95% CI)** | **LR(-)**  **(95% CI)** | **OR^*^**  **(95% CI)** | **AUC** ^¶^  **(95% CI)** |
| **Overall** | 8.45 (8.44 - 8.46) | 0.40 (0.39 - 0.40) | 21.39 (21.33 - 21.44) | 0.78 (0.78 - 0.78) |  | 6.50 (6.49 - 6.52) | 0.70 (0.70 - 0.70) | 9.27 (9.24 - 9.29) | 0.64 (0.64 - 0.64) |
| **Age group (y)** |  |  |  |  |  |  |  |  |  |
| 65-74 | 10.80 (10.77 - 10.83) | 0.47 (0.47 - 0.47) | 22.92 (22.79 - 23.05) | 0.75 (0.75 - 0.75) |  | 5.02 (5.00 - 5.04) | 0.82 (0.82 - 0.82) | 6.14 (6.12 - 6.17) | 0.59 (0.59 - 0.59) |
| 75-84 | 7.04 (7.03 - 7.06) | 0.39 (0.39 - 0.39) | 18.12 (18.05 - 18.20) | 0.78 (0.78 - 0.78) |  | 6.66 (6.64 - 6.68) | 0.66 (0.66 - 0.66) | 10.07 (10.03 - 10.10) | 0.66 (0.66 - 0.66) |
| ≥85 | 5.35 (5.34 - 5.37) | 0.39 (0.39 - 0.39) | 13.86 (13.79 - 13.93) | 0.77 (0.77 - 0.77) |  | 4.80 (4.78 - 4.82) | 0.68 (0.67 - 0.68) | 7.11 (7.08 - 7.15) | 0.65 (0.65 - 0.65) |
| **Sex** |  |  |  |  |  |  |  |  |  |
| Male | 7.53 (7.51 - 7.55) | 0.50 (0.50 - 0.50) | 14.99 (14.92 - 15.05) | 0.73 (0.73 - 0.73) |  | 4.91 (4.89 - 4.92) | 0.79 (0.79 - 0.79) | 6.22 (6.20 - 6.25) | 0.60 (0.60 - 0.60) |
| Female | 8.78 (8.76 - 8.79) | 0.34 (0.34 - 0.34) | 25.96 (25.87 - 26.05) | 0.80 (0.80 - 0.81) |  | 7.74 (7.72 - 7.76) | 0.63 (0.63 - 0.64) | 12.19 (12.15 - 12.22) | 0.67 (0.67 - 0.67) |
| **Race and ethnicity** |  |  |  |  |  |  |  |  |  |
| White, non-Hispanic | 8.96 (8.94 - 8.97) | 0.38 (0.38 - 0.38) | 23.63 (23.56 - 23.71) | 0.79 (0.79 - 0.79) |  | 6.87 (6.86 - 6.89) | 0.69 (0.69 - 0.69) | 9.97 (9.95 - 10.00) | 0.65 (0.65 - 0.65) |
| Black, non-Hispanic | 6.16 (6.14 - 6.19) | 0.40 (0.40 - 0.40) | 15.44 (15.32 - 15.56) | 0.77 (0.77 - 0.77) |  | 7.64 (7.58 - 7.69) | 0.65 (0.65 - 0.65) | 11.72 (11.63 - 11.81) | 0.67 (0.67 - 0.67) |
| Hispanic | 6.47 (6.44 - 6.50) | 0.35 (0.35 - 0.35) | 18.37 (18.22 - 18.53) | 0.79 (0.79 - 0.79) |  | 3.08 (3.06 - 3.10) | 0.77 (0.77 - 0.77) | 4.00 (3.97 - 4.03) | 0.60 (0.60 - 0.60) |
| Other, non-Hispanic | 18.84 (18.61 - 19.08) | 0.58 (0.58 - 0.58) | 32.55 (32.08 - 33.02) | 0.71 (0.70 - 0.71) |  | 14.18 (13.91 - 14.46) | 0.81 (0.81 - 0.82) | 17.40 (17.06 - 17.75) | 0.59 (0.59 - 0.59) |
| **Education** |  |  |  |  |  |  |  |  |  |
| Less than high school | 4.33 (4.32 - 4.34) | 0.43 (0.43 - 0.43) | 10.15 (10.10 - 10.19) | 0.74 (0.74 - 0.75) |  | 3.12 (3.11 - 3.13) | 0.72 (0.72 - 0.72) | 4.33 (4.32 - 4.35) | 0.62 (0.62 - 0.62) |
| High school graduate | 10.23 (10.20 - 10.25) | 0.33 (0.33 - 0.33) | 30.84 (30.69 - 30.99) | 0.81 (0.81 - 0.81) |  | 8.39 (8.36 - 8.42) | 0.67 (0.67 - 0.67) | 12.49 (12.44 - 12.54) | 0.66 (0.66 - 0.66) |
| Some college | 9.60 (9.56 - 9.64) | 0.39 (0.39 - 0.40) | 24.38 (24.20 - 24.56) | 0.78 (0.78 - 0.78) |  | 5.61 (5.58 - 5.63) | 0.74 (0.74 - 0.75) | 7.52 (7.48 - 7.57) | 0.62 (0.62 - 0.62) |
| College graduate and higher | 10.24 (10.19 - 10.29) | 0.58 (0.58 - 0.58) | 17.62 (17.49 - 17.76) | 0.70 (0.70 - 0.70) |  | 7.53 (7.49 - 7.57) | 0.79 (0.79 - 0.79) | 9.51 (9.45 - 9.56) | 0.60 (0.60 - 0.60) |
| **Abbreviations:** SCI, subjective cognitive impairment; CI, confidence interval; y, years; ROC, receiver operator characteristic; OR, odds ratio; AUC, area under the Receiver operator characteristic (ROC) curve.  **Notes:** We assume that the NHATS probable dementia definition to be the reference standard for dementia identification, as it is widely used in the literature to quantify dementia prevalence. We then assess the performance of the SCI measure against this NHATS probable dementia definition to assess the validity of SCI as a population-based dementia risk identification tool. Sample included n=1,936 observations (weighted n=35,489,497). All estimates are adjusted using Round 2 analytic weights, to produce a nationally representative sample and account for differential probabilities of selection and nonresponse in NHATS and were further adjusted to reflect the fact that the analytic sample is a random one-third sample of NHATS participants in 2012.  ^*^ The odds ratio (OR) is the ratio of the odds of reporting having SCI among individuals with dementia (as assessed by the NHATS probable dementia definition), to the odds of reporting having SCI among individuals without dementia.  ^¶^ The AUC is the area under the Receiver operator characteristic (ROC) curve and measures the performance of SCI in correctly identifying individuals with dementia and those without dementia. ROC curves graphically relate true positive rates (sensitivity) to false negative rates (1- specificity) across a range of values. | | | | | | | | | |

**References**

1. Galvin JE, Roe CM, Powlishta KK, et al. The AD8: A brief informant interview to detect dementia. *Neurology*. Aug 23 2005;65(4):559-64. doi:10.1212/01.wnl.0000172958.95282.2a

2. Kasper JD, Freedman VA, Spillman B. Classification of Persons by Dementia Status in the National Health and Aging Trends Study. Technical Paper #5. Baltimore: Johns Hopkins University School of Public Health; 2013.

3. Centers for Disease Control and Prevention. Behavioral Risk Factor Surveillance System: Complex sampling weights and preparing 2017 BRFSS module data for analysis. 2019.

4. Centers for Disease Control and Prevention. Complex sampling weights and preparing 2018 BRFSS module data for analysis. 2019.

5. Luo H, Yu G, Wu B. Self-Reported Cognitive Impairment Across Racial/Ethnic Groups in the United States, National Health Interview Survey, 1997-2015. *Prev Chronic Dis*. Jan 11 2018;15:E06. doi:10.5888/pcd15.170338

6. Bernstein AB, Remsburg RE. Estimated prevalence of people with cognitive impairment: results from nationally representative community and institutional surveys. *Gerontologist*. Jun 2007;47(3):350-4. doi:10.1093/geront/47.3.350

7. National Center for Health Statistics. NHIS Data, Questionnaires and Related Documentation. Feb 26, 2021. Accessed February 26, 2021. <https://www.cdc.gov/nchs/nhis/data-questionnaires-documentation.htm>

8. Alzheimer’s Disease Working Group (ADWG). *Washington State Plan to Address Alzheimer's Disease and Other Dementias*. 2016. Accessed July 12, 2021. <https://act.alz.org/site/DocServer/WA_State_Plan_Jan_2016_.pdf;jsessionid=00000000.app20041a?docID=50669&NONCE_TOKEN=F1F4E5508E225FC86080AB63C5F7069F>

9. Taylor CA, Bouldin ED, McGuire LC. Subjective cognitive decline among adults aged≥ 45 years—United States, 2015–2016. *Morbidity and Mortality Weekly Report*. 2018;67(27):753.

10. Landis JR, Koch GG. The Measurement of Observer Agreement for Categorical Data. *Biometrics*. 1977;33(1):159-174. doi:10.2307/2529310

11. Florkowski CM. Sensitivity, specificity, receiver-operating characteristic (ROC) curves and likelihood ratios: communicating the performance of diagnostic tests. *Clin Biochem Rev*. 2008;29 Suppl 1(Suppl 1):S83-S87.

12. Seed P. DIAGT: Stata module to report summary statistics for diagnostic tests compared to true disease status. 2010;

13. Hosmer DW, Lemeshow S, Sturdivant RX. *Applied logistic regression*. Wiley New York; 2000.

14. CLSI. User protocol for evaluation of qualitative test performance; approved guideline. Clinical and Laboratory Standards Institute Wayne (PA); 2008.
